# Dynamics of Viral and Host Immune Cell MicroRNA Expression during Acute Infectious Mononucleosis

**DOI:** 10.3389/fmicb.2017.02666

**Published:** 2018-01-15

**Authors:** Vandana Kaul, Kenneth I. Weinberg, Scott D. Boyd, Daniel Bernstein, Carlos O. Esquivel, Olivia M. Martinez, Sheri M. Krams

**Affiliations:** ^1^Division of Abdominal Transplantation, Department of Surgery, Stanford University, Stanford, CA, United States; ^2^Division of Stem Cell Transplantation, Department of Pediatrics, Stanford University, Stanford, CA, United States; ^3^Department of Pathology, Stanford University, Stanford, CA, United States; ^4^Division of Cardiology, Department of Pediatrics, Stanford University, Stanford, CA, United States; ^5^Stanford Immunology, Stanford University School of Medicine, Stanford, CA, United States

**Keywords:** RNA, acute infectious mononucleosis, Epstein–Barr virus, immunology

## Abstract

Epstein–Barr virus (EBV) is the etiological agent of acute infectious mononucleosis (IM). Since acute IM is a self-resolving disease with most patients regaining health in 1–3 weeks there have been few studies examining molecular signatures in early acute stages of the disease. MicroRNAs (miRNAs) have been shown, however, to influence immune cell function and consequently the generation of antibody responses in IM. In this study, we performed a comprehensive analysis of differentially expressed miRNAs in early stage uncomplicated acute IM. miRNAs were profiled from patient peripheral blood obtained at the time of IM diagnosis and at subsequent time points, and pathway analysis performed to identify important immune and cell signaling pathways. We identified 215 differentially regulated miRNAs at the most acute stage of infection when the patients initially sought medical help. The number of differentially expressed miRNAs decreased to 148 and 68 at 1 and 2 months post-primary infection, with no significantly changed miRNAs identified at 7 months post-infection. Interferon signaling, T and B cell signaling and antigen presentation were the top pathways influenced by the miRNAs associated with IM. Thus, a dynamic and regulated expression profile of miRNA accompanies the early acute immune response, and resolution of infection, in IM.

## Introduction

Epstein–Barr virus is a gamma-herpes family virus that infects over 95% of humans globally and is the etiological agent of many acute and chronic infections as well as human malignancies (Khan and Hashim, 2014). While EBV infection is widespread, the outcome of infection can depend upon the age of the host. Pediatric cases are mainly asymptomatic and the infection subsides due to a vigorous host T cell response with the virus subsequently transitioning to latency in a subset of memory B cells (Jayasooriya et al., 2015; Gantt et al., 2016). However, in adolescents, primary infection can manifest as glandular fever, lymphadenopathy, and a sore throat, termed infectious mononucleosis (IM) (Balfour et al., 2013). IM is associated with a transient proliferation of EBV-infected B cells followed by a considerable expansion of EBV-specific T cells (Sixbey et al., 1984; Kurth et al., 2000; Chaganti et al., 2009; Hadinoto et al., 2009; Balfour et al., 2013). IM is typically self-limiting and resolves over a period of weeks to months with the virus persisting into a latent phase in infected B cells (Hochberg et al., 2004).

MicroRNAs are small 18–25 nt long single-stranded RNA molecules which regulate gene expression at the post-transcriptional level. miRNA target specific mRNAs by complementary base-pairing, leading to cleavage or repression of translation, and can act as major regulators of key cellular processes. We (Harris et al., 2010) and others (Forte et al., 2012; Mansouri et al., 2014) have shown that EBV infection can alter the expression of host miRNAs and thus modulate various arms of the cellular machinery including cell differentiation and death, proliferation, and the immune response. Along these lines, miR-194 is suppressed by EBV and participates in regulation of IL-10 expression in EBV lymphomas (Harris et al., 2010) while two miRNA families, the let-7 family and the miR-200 family, as well as miR-143-3p, act as tumor-suppressor miRNAs and are significantly downregulated in EBV-infected gastric carcinoma cells (Marquitz et al., 2014). Other studies have found that miR-155 and miR-21 were induced in B cells by EBV infection and potentially play a role in viral tumorigenesis (Linnstaedt et al., 2010; Rosato et al., 2012; Anastasiadou et al., 2015). Furthermore, p53-targeted tumor suppressor, miR-34a, is strongly induced by EBV infection in many EBV and Kaposi’s sarcoma-associated herpesvirus-infected lymphoma cell lines and plays a role in B cell transformation (Forte et al., 2012). In addition, miRNAs have been identified that play a critical role in the T cell response to pathogens. For example, miR-155 is essential for the CD8^+^ effector T cell response to mCMV (Dudda et al., 2013).

In addition to host miRNAs, EBV-expressed miRNAs have also been shown to modulate the function of T and B cells during an immune response. In a study by Albanese et al. (2016), EBV-encoded miRNAs were found to inhibit CD8^+^ T cell-mediated viral surveillance at multiple levels. The viral miRNAs downregulate TAP-complex and HLA allotype that present TAP-dependent epitopes, reduce IL-12 secretion by infected B cells to diminish EBV-specific CD8 T cells and repress EBNA1, a viral protein responsible for latency (Albanese et al., 2016). EBV encoded miRNAs miR-BART1, miR-BART2, and miR-BHRF1-2 have also been shown to target the host adaptive immune response by repressing IL-12 secretion resulting in reduced differentiation of CD4^+^ T cells into the Th1 subset and inhibiting lysosomal enzymes involved in MHC class-II peptide processing (Tagawa et al., 2016), while miR-BART16 suppresses Type I IFN signaling (Hooykaas et al., 2017). In the current study, we report the differential expression of host and viral miRNAs in acute IM caused by primary EBV infection. Our findings elucidate the miRNA signature associated with acute IM and demonstrate the dynamic features of this response with the miRNA expression profile reverting to that of healthy individuals within a few months of IM diagnosis in subjects without complications.

## Materials and Methods

### Patient Samples

Peripheral blood samples were obtained from symptomatic patients (aged 18–24 years) who visited Vaden Student Health Center at Stanford University and were diagnosed with clinical IM. Samples were obtained upon diagnosis (time point 0) and then at, 1, 2, and 7 months. Blood samples were processed using Ficoll-Paque PLUS (Amersham Pharmacia Biotech, Piscataway, NJ, United States) then PBMC frozen and maintained in liquid nitrogen until use. PBMCs were isolated from blood derived from healthy age-matched individuals. This study was carried out in accordance with the recommendations of Institutional Review Board at Stanford University with written informed consent from all subjects. All subjects gave written informed consent in accordance with the Declaration of Helsinki.

### RNA Isolation and miRNA Microarray

Patient (*n* = 16) and healthy control (*n* = 3) PBMCs were suspended and lysed in TRIzol Reagent (Life Technologies, Foster City, CA, United States) for isolation of total RNA. Glycogen (20 μg) was used for RNA precipitation. Isolated RNA was DNase treated (Qiagen) and clean-up was done on RNeasy spin columns (Qiagen) using a modified protocol which replaced RW1 buffer with RWT buffer for washing. RNA was suspended in RNase free water and stored at -80°C. RNA quantification was done using Nanodrop 2000 Spectrophotometer (Thermo Fisher scientific, Wilmington, DE, United States). Approximately 200 ng RNA was used for microarray analysis using Affymetrix GeneChip miRNA 4.0 arrays (Affymetrix, Santa Clara, CA, United States) through the PAN (Protein and Nucleic acid) facility at Stanford University. Briefly, 130 ng total RNA was assessed for quality using Bioanalyzer 2100 (Agilent), followed by Poly-A tailing and labeling with biotin using a Affymetrix FlashTag Biotin HSR RNA Labeling kit (Affymetrix, Santa Clara, CA, United States) according to the manufacturer’s protocol. The labeled targets are hybridized to the GeneChip^®^ miRNA 4.0 arrays per standard miRNA protocol, which includes 16 h (overnight) hybridization at 48°C at 60 RPM in the Affymetrix GeneChip Hybridization oven 645. The arrays were then washed and stained in the Affymetrix GeneChip Fluidics Station 450. The arrays were scanned using the Affymetrix GeneChip Scanner 3000 7G. The GeneChip^®^ miRNA 4.0 arrays contain 30,424 total mature miRNA probe sets including 2578 mature human miRNAs, 2025 pre-miRNA human probes, 1996 Human snoRNA and scaRNA probe sets and miRNAs of 202 other organisms.

### Data Analysis

Raw microarray data were statistically analyzed using Partek Genomics Suite software (Partek Inc.). Normalization was done suing Robust Multi-array Average (RMA) and log2 transformation of data was performed, followed by one-way ANOVA statistical testing to identify significantly differentially expressed miRNA between different groups (*P* ≤ 0.05). After running ANOVA, the software performs multi-testing correction using Benjamini–Hochberg Step-Up FDR-controlling procedure for all the expressed miRNAs (default FDR Alpha level 0.05). Venn diagram depicting intersection of differentially expressed miRNAs and Unsupervised Hierarchical Clustering analysis was also performed. Raw Fold change and adjusted *p*-value data was exported to dot excel file and data selection and graphic visualization was done using Power BI software (Microsoft Inc.). Differentially expressed EBV miRNAs were also identified using the same miRNA microarray dataset using the miRNA filter as “EBV,” mRNA expression data from a publicly available GEO dataset (GSE45924) was used to perform miRNA–mRNA integrated analysis. Briefly, SOFT formatted family file (GSE45924-GPL6883_series_matrix.piv.txt.fmt) were downloaded from the GEO Database and imported into Partek Genomics Suite for mRNA “Gene Expression” workflow. Illumina HumanRef-8 v3.0 expression beadchip annotation files were uploaded to the Partek Software to obtain Fold Change Values for mRNA differential expression between comparison groups of Acute IM subjects (PBMC) and control subjects (PBMC). Pre-normalized data was log2 transformed, followed by one-way ANOVA statistical testing to identify significantly differentially expressed mRNAs and multiple-testing correction using Benjamini–Hochberg Step-Up FDR-controlling procedure (default FDR Alpha level 0.05). Fold increases of >2, and fold decreases of >2 were considered significant. The differentially expressed mRNA data was exported into a text file and converted into a suitable format for upload to IPA for further analysis.

### Functional and Pathway Enrichment Analysis of Target Genes Controlled by Differentially Expressed miRNAs

Pathway and molecular function enrichment analysis was performed using IPA software (Qiagen, Redwood city, CA, United States). A list of all twofold differentially expressed miRNAs obtained from Partek Genomics Suite software analysis was uploaded into IPA for analysis. miRNA target enrichment was performed using the “miRNA Target Filter” function, generating a list of 181 miRNAs with available targeting information. An mRNA gene expression dataset (GSE45924) obtained from GEO database was added to the “miRNA Target Filter” setting in IPA and “Expression pairing” was performed. An additional filter applied to this list was “Applied filters: confidence = Experimentally Observed OR High (predicted).” Further “Core Analysis” was performed on the target mRNAs based on different experimental time points to determine the affected networks and canonical pathways. Throughout the analysis, a *p*-value cutoff of 0.05 was applied. The HumanRef-8 v3.0 reference gene list was used to set the population of genes to consider for *p*-value calculations while performing Core Analysis.

## Results

### Sample QC and Principal Component Analysis

**FIGURE 1:**
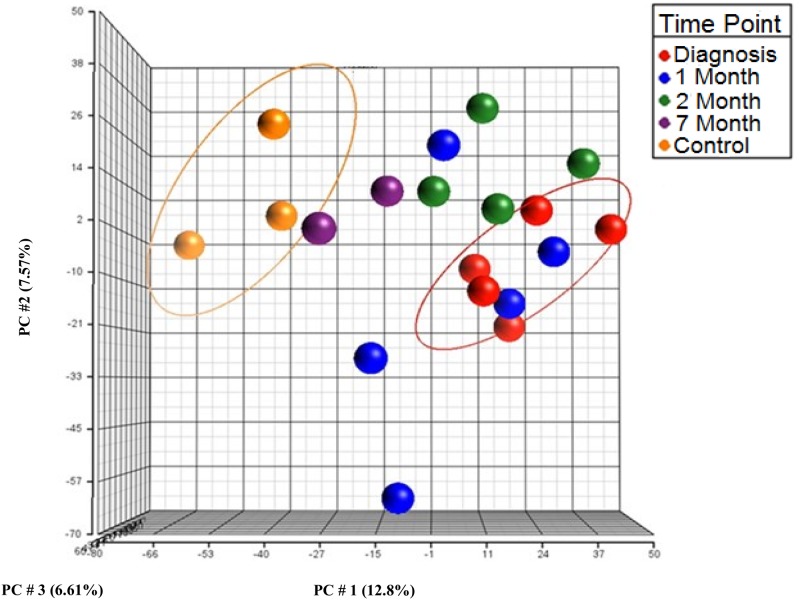
Principal component analysis (PCA) on miRNA expression data from human peripheral blood mononuclear cells (PBMCs) from acute infectious mononucleosis (IM) patients and healthy controls using the first three components. Red, at diagnosis; Blue, 1 month; Green, 2 months; Purple, 7 months; Orange, Healthy control.

As a means of quality control to determine the biological separation of the groups based on probe intensities, principal component analysis (PCA) was performed using Partek Genomics software. Two separate groups of samples from IM patients at 0 month (at the time of diagnosis) and healthy controls are clearly observed (**Figure 1**). The remaining samples from 1, 2, and 7 months are loosely segregated into groups and fall between these two groups on PC1 with the samples obtained at 7 months grouping closely with those from healthy controls. These findings indicate that the differentially expressed miRNAs are maximal at the time of IM diagnosis and return to the healthy control pattern in subsequent months.

### Differentially Expressed miRNAs

**FIGURE 2:**
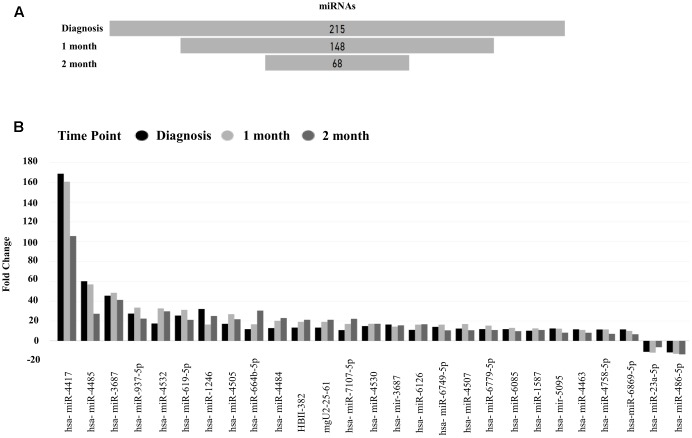
Top differentially expressed microRNAs (miRNAs). **(A)** Differentially expressed miRNAs in IM Patients at Time 0, 1, and 2 months compared to healthy controls, **(B)** Top differentially expressed miRNAs observed at all three time points, at diagnosis, 1, and 2 months vs. healthy controls [false discovery rate (FDR) 0.05, Absolute Fold change >10].

The differential expression of miRNAs were compared between patients at the time of IM diagnosis (0 month), and at 1 and 2 months after diagnosis, compared to age-matched healthy controls (**Figure 2A**). We identified 215 differentially regulated miRNAs at the most acute stage of infection (diagnosis, 0 month). The number of differentially expressed miRNAs decreased to 148 and 68 during the course of 1 and 2 months post-primary infection, with no significantly changed miRNAs identified at 7 months post-infection (data not shown). The 27 top differentially expressed miRNAs at diagnosis, and 1 and 2 months after diagnosis, show that most of the differentially expressed miRNAs are increased (25 increased, 2 decreased) (**Figure 2B**).

**FIGURE 3:**
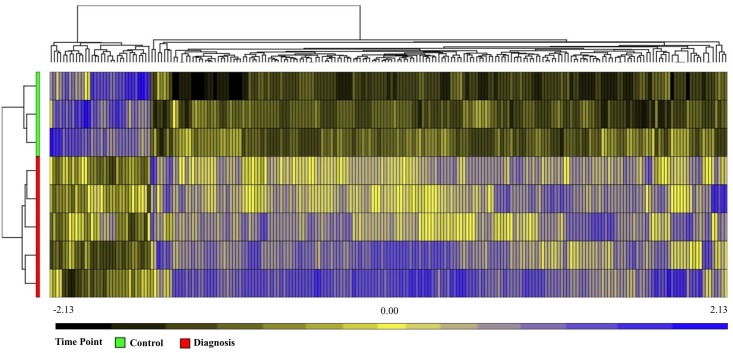
Unsupervised Hierarchical Clustering Heatmap showing relative intensity for 215 miRNAs in Patients at diagnosis versus healthy controls. Analysis using Partek Genomics suite, step up FDR 0.05. Black shows downregulation and blue shows upregulation. Healthy controls (green), IM at diagnosis (red).

**FIGURE 4:**
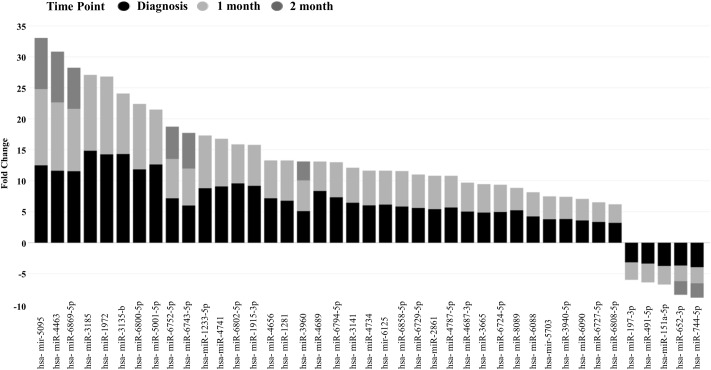
Differentially expressed miRNAs at three time points, at Diagnosis, 1 and 2 months showing a trend toward decreasing absolute fold change (FDR 0.05).

**FIGURE 5:**
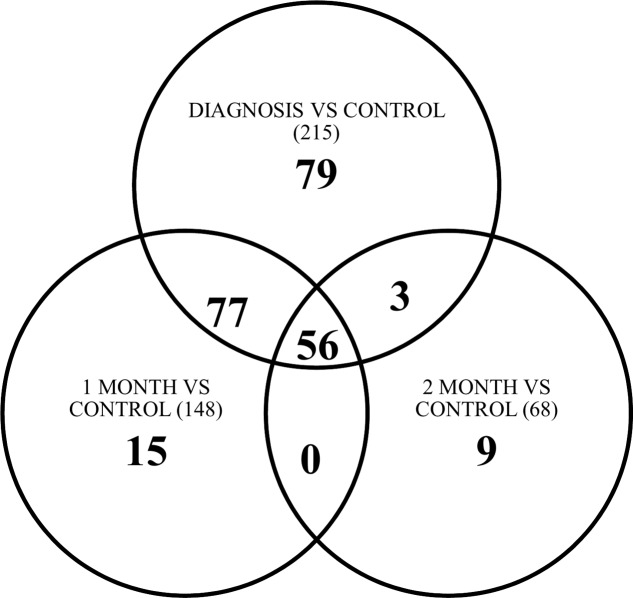
Venn diagram depicting number of miRNAs common between point of diagnosis, 1 and 2 months versus healthy control. Number in brackets () is the total number of differentially expressed miRNAs at that time point vs. control.

Unsupervised hierarchical clustering depicted as a heatmap shows the relative intensity for the 215, 148, and 68 differentially expressed miRNAs at diagnosis (**Figure 3**), 1 month (Supplementary Figure S1) and 2 months (Supplementary Figure S2) as compared to healthy controls, respectively. A fold change of two and FDR adjusted *p*-value (Benjamini–Hochberg method for multiple corrections) of 0.05 was considered significant in Partek Genomics Suite Software, with black depicting decreased expression levels and blue depicting upregulation. Furthermore, the differentially expressed miRNAs demonstrate a decreasing absolute fold change over time, with the highest fold change generally observed at the time of diagnosis (**Figure 4**). These 41 miRNAs exhibit a dynamic differential expression pattern that trends toward healthy control levels at 2 months post IM diagnosis. Two miRNAs that are highly upregulated, miR-4417 and miR-4485 (data not shown), exhibit a similar trend of decreasing differential expression from diagnosis to 2 months. A compilation of all statistically significant differentially expressed miRNAs at diagnosis and one, and 2 months versus healthy controls is included in Supplementary Table S1. Supplementary Table S2 provides a list of all differentially expressed miRNAs. A Venn diagram depicts the number of miRNAs in common at IM diagnosis and at 1 and 2 months (**Figure 5**). The intersection shows that 56 miRNAs are differentially expressed at the three time points, thus providing important information regarding possible disease specific signatures. Interestingly, miRNAs uniquely expressed at diagnosis, 1 and 2 months after diagnosis also decrease in number during the course of the infection toward recovery (79 at diagnosis, 15 at 1 month and 9 at 2 months).

### Pathways Altered by IM

**FIGURE 6:**
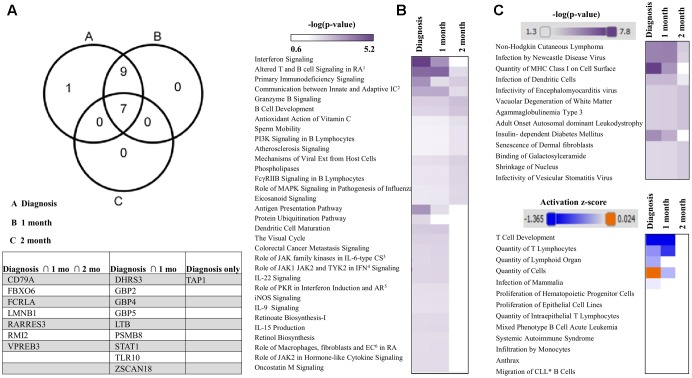
mRNA targets and associated pathways of the differentially expressed miRNAs. **(A)** Venn diagram and table comparison of mRNA targets of differentially expressed miRNAs at Diagnosis, 1 and 2 months. **(B)** Comparison of canonical pathways, **(C)** Comparison of Diseases and Bio Functions; mRNA targets were obtained from miRNA–mRNA expression pairing of differentially expressed miRNAs and GEO dataset GSM1119621–GSM1119636 in ingenuity pathway analysis (IPA).

Expression pairing of miRNA data, from our study and gene expression data from GEO set GSE45924 returned 46 miRNAs targeting 17 mRNAs (**Figure 6A**). Comparative analysis of mRNA targets highlighted the most canonical pathways as well as disease and biological functions (**Figures 6B,C**). Furthermore, functional pathway analysis in IPA using the list of differentially expressed miRNAs at 0 month suggests the most impacted networks include antigen presentation, infectious disease, humoral immune response, protein synthesis, cell cycle, and cell death mechanisms. Networks have been shown in addition to their scores and number of mRNA targets (including particular molecules that pass our set filter criterion) (**Table 1**). Further analysis in IPA for top canonical pathways targeted by our list of differentially expressed miRNAs at IM diagnosis and based on the Illumina HumanRef-8 v3.0 gene superset shows candidates including interferon signaling, altered T and B cell signaling, primary immunodeficiency signaling, antigen presentation and communication between innate and adaptive immune cells as the major affected canonical pathways (Supplementary Figure S3).

**Table 1:** List of top five networks with their respective scores obtained from ingenuity pathway analysis (IPA): Data are indicative of networks regulated by mRNA targets of microRNAs (miRNAs) differentially expressed at the time of infectious mononucleosis (IM) diagnosis.

Top diseases and functions	Score	Focus molecules^∗^	Molecules in network
Antigen presentation, protein synthesis, infectious disease	36	13	BCR (complex), **↓CD79A**, ERK1/2, **↑FBX06**, **↓FCRLA**, **↑GBP2**, **↑GBP4**, **↑GBP5,** IFNα, β, IFN type1, Ifnar, Ifnz, IgG, IgM, IL12 (family), **↑LMNB1**, **↓LTB**, MHC Class I (complex), NF-κB (complex) Nr1hPirb, **↑PSMB8**, **↑RARRES3,** RNA polymerase II, SLFN13, **↑STAT1**, **↑TAP1**, TBCB, TCR, **↓TLR10**, Trbv1
Hematological system development and function, humoral immune response, lymphoid tissue structure and development	3	1	FOXO1, IGHM, KLF3, SOX11, **↓VPREB3**
Cell morphology, cellular function and maintenance, connective tissue disorders	2	1	APP, HNF4A, HOXD4, LIG4, SAFB2, ZNF24, ZNF396, ZSCAN1, ZSCAN18, ZSCAN20, **↓ZSCAN32**
Cell cycle cellular assembly and organization, DNA replication, recombination and repair	2	1	BLM, CCND1, CENPS/CENPS-CORT, dihydrotestosterone, ERCC6L, FAAP100, FANCA, FANCM, NOTCH3, RIF3, RMI1, **↑RMI2**, RPA1, RPA2, RPA3, SPIDR, TOP3A, TOPBP1, TTK
Cell cycle, gene expression, cell death and survival	2	1	All *trans*-retinyl esters, CREBBP, **↓DHRS3**, ELAVLi, EP300, FSH, GPS2, Lh, N-cor, NADP-retinol dehydrogenase, OSM, PLA2G10, PPARG, RAF1, RDH, RDH10, retinyl ester, SNCA, TNF, TP73, tretinoin


*^∗^Focus Molecules (in bold)- molecules that passed the filters and cut-offs that are members of this network.*

### Differentially Expressed EBV miRNAs

Interestingly, when the EBV miRNAs are analyzed using specific filtering criteria in Partek, only ebv-miR-BART-16 and ebv-miR-BART5-3p are uniquely upregulated at IM diagnosis and at 1 and 2 months as compared to healthy controls (ANOVA 0.05, unadjusted *p*-value) (**Table 2**). It is notable that no differential expression of any EBV miRNAs is observed at 7 months post-infection, which is expected due to resolution of the symptoms in the acute phase and establishment of EBV latency. Thus, of the more than 40 known EBV miRNAs only two, miR-BART5-3p and miR-BART-16 are expressed during acute IM (Skinner et al., 2017).

**Table 2:** Differentially expressed EBV miRNAs in patients at diagnosis, 1 and 2 months versus healthy controls, ANOVA 0.05, unadjusted *p*-value.

	0 Month (diagnosis)			1 Month			2 Months		
								
EBV miRNA	Fold change	*p*-value	↑/↓	Fold change	*p*-value	↑/↓	Fold change	*p*-value	↑/↓
ebv-miR-BART16	2.93	0.00	↑	2.75	0.01	↑	2.11	0.04	↑
ebv-miR-BART5-3p	2.44	0.02	↑	2.57	0.01	↑	2.85	0.01	↑


## Discussion

This study provides the first miRNA expression profile of sequential, peripheral blood samples from college-age patients with acute IM. Our results clearly indicate that primary EBV disease is accompanied by marked expression changes of multiple host and viral miRNA, and that the number of differentially expressed miRNAs decreases substantially after the initial diagnosis. It is noteworthy that there were no differentially expressed miRNAs observed at 7 months as compared to healthy controls, indicating that there is no identifiable miRNA signature that can distinguish between normal college students and those that have resolved IM. Since none of the patients studied had any complications after IM, it remains possible that expression of the differentially expressed host miRNAs would remain dysregulated if IM were not completely resolved.

We determined that 43 of the host miRNAs, including miR-197-3p, miR-491-5p, miR-151a-5p, miR-652-3p, and miR-744-5p, decreased from the levels found at diagnosis over the subsequent 2 months. While the specific function of these miRNA in IM is unknown, other reports have linked these miRNA to disease processes. Recently, miR-197 was found to act synergistically with EBV-BART6-3p to reduce the expression of IL-6R, thereby compromising host immune defense in EBV-positive Burkitt Lymphoma (Zhang et al., 2017). miR-625-3p has been shown to be a potential biomarker for a number of cancerous conditions including malignant pleural mesothelioma (Kirschner et al., 2012). miR-625-3p targets p38 MAPK activator MAP2K6 and induces oxaliplatin resistance by abrogating MAP2K6-p38-regulated apoptosis and cell cycle control networks (Rasmussen et al., 2016). A recent study also shows that miR-625-3p is upregulated in CD8^+^ T cells during early immune reconstitution following allogeneic stem cell transplantation (Verma et al., 2017). An interesting study with highly virulent H5N2 strain of mouse-adapted avian influenza virus found miR-151a-p as one of the significantly upregulated miRNAs, whose inhibition resulted in a 70% reduction in mortality of inoculated mice (Choi et al., 2014).

The majority of studies to date examining the impact of EBV infection or pathogenesis on miRNA expression have focused on the miRNA profile within the virally infected cell and the implications for tumorigenesis. In contrast, we analyzed the impact of EBV infection on peripheral lymphoid cells that typically participate in the immune response to EBV. A single previous study did examine the cellular miRNAs from pre-adolescent children with IM in China over the first 14 days after primary infection (Gao et al., 2015). They determined the host miRNA expression in B cells and CD8^+^ T cells and EBV miRNAs in plasma and B cells. Interestingly, some miRNAs such as miR-155 and miR-142 were increased in B cells and decreased in CD8^+^ T cells, thus, changes would not be reflected in unfractionated PBMC. Our analysis of miRNAs in PBMC demonstrated a different set of host miRNAs as differentially expressed. These distinct patterns are most likely attributed to different methodologies, since our study took an unbiased approach and performed microarray analysis using Affymetrix GeneChip miRNA 4.0 arrays (over 30,000 mature miRNAs) on unfractionated PBMC, whereas the Gao group specifically examined a small subset of 84 miRNAs in sorted B and T cells via PCR. Further, the ages of the subjects were different and there could be other differences including environmental factors and variant strains of EBV. EBV expresses more miRNAs than other characterized human viruses, with at least 44 EBV miRNAs identified to date (Skinner et al., 2017). We determined that ebv-miR-BART16 and ebv-miR-BART5-3p were increased in PBMC as compared to healthy controls. The specific function of these viral miRNA in IM is unknown, but it is interesting that a recent report indicates that BART16 suppresses IFN signaling (Hooykaas et al., 2017). Notably, the EBV miRNA profile we identified in acute IM is distinct from the EBV miRNA profile identified in plasma samples of patients with chronic active EBV infection (Kawano et al., 2013).

In addition to EBV-induced miRNA regulation in infected B cells, others have focused on understanding the EBV pathogenesis and host responses by transcriptomic (mRNA microarray, Illumina) analysis on PBMCs of human subjects in a prospective study of naturally acquired primary EBV infection (Dunmire et al., 2014). The authors found a distinct gene expression profile in acute but not latent EBV infection. Moreover, upon gene expression profiling in sorted CD8^+^ T cells, NK cells, monocytes, and B cells, it was found that CD8^+^ T cells and monocytes showed upregulation of key gene groups (Type I interferon regulated genes (IRGs), type II IRGs, and cell cycle/metabolism genes.) during acute infection. This pathway analysis agrees with the pathways predicted to be altered by our miRNA profile during acute IM.

## Conclusion

We have defined the host immune cell and EBV miRNA profile elicited by primary EBV infection during acute IM. This profile is indicative of a response that is accompanied by complete resolution of disease. Thus, it may be useful in predicting which individuals that present with IM will have an uncomplicated course and ultimately may shed insight into the host immune response to acute EBV infection.

## Author Contributions

VK designed and performed experiments, analyzed the data, and wrote the manuscript. KW obtained the samples and contributed to development of study design. SB contributed to development of study design and critically revised the manuscript. DB contributed to development of study design. CE contributed to development of study design. OM contributed to study design, provided critical review of the experimental design, wrote, and provided critical review of the manuscript. SK designed the study, analyzed the data, wrote, and critically reviewed the manuscript. All authors reviewed and approved the final manuscript.

## Conflict of Interest Statement

The authors declare that the research was conducted in the absence of any commercial or financial relationships that could be construed as a potential conflict of interest.

## Abbreviations

EBV: Epstein–Barr virus
FDR: false discovery rate
IPA: ingenuity pathway analysis
miRNA: microRNA
PBMC: peripheral blood mononuclear cells
