# Supplementary material for: Dynamics of Viral and Host Immune Cell MicroRNA Expression during Acute Infectious Mononucleosis
Source: Front Microbiol. 2018 Jan 15;8:2666. doi: 10.3389/fmicb.2017.02666 (PMC5775229; doi:10.3389/fmicb.2017.02666)
Supplement: Supplementary file 1 [file Presentation_1.pdf]

Supplementary information

1. GEO dataset analysis in Partek Genomics Suite

Downloaded files: GSE45924-GPL6883_series_matrix.piv.txt.fmt from GEO

Reference: Dunmire, S.K., Odumade, O.A., Porter, J.L., Reyes-Genere, J., Schmeling, D.O., Bilgic, H., Fan, D., Baechler, E.C., Balfour, H.H., Hogquist, K.A., et al. (2014). Primary EBV infection induces an expression profile distinct from other viruses but similar to hemophagocytic syndromes. PLoS One *9*, e85422.

Platform used: GPL6883-Illumina HumanRef-8 v3.0 expression beadchip

Samples: 8 acute IM and 8 seronegative subjects

GSM1119621 PBMC Acute IM Subj 5027 (Ref8)

GSM1119622 PBMC Seronegative Subj 5027 (Ref8)

GSM1119623 PBMC Acute IM Subj 5036 (Ref8)

GSM1119624 PBMC Seronegative Subj 5036 (Ref8)

GSM1119625 PBMC Acute IM Subj 5088 (Ref8)

GSM1119626 PBMC Seronegative Subj 5088 (Ref8)

GSM1119627 PBMC Acute IM Subj 5324 (Ref8)

GSM1119628 PBMC Seronegative Subj 5324 (Ref8)

GSM1119629 PBMC Acute IM Subj 5342 (Ref8)

GSM1119630 PBMC Seronegative Subj 5342 (Ref8)

GSM1119631 PBMC Acute IM Subj 5483 (Ref8)

GSM1119632 PBMC Seronegative Subj 5483 (Ref8)

GSM1119633 PBMC Acute IM Subj 5509 (Ref8)

GSM1119634 PBMC Seronegative Subj 5509 (Ref8)

GSM1119635 PBMC Acute IM Subj 5524 (Ref8)

GSM1119636 PBMC Seronegative Subj 5524 (Ref8)

Filters used to analyze mRNA GEO data in Partek Genomics Suite analysis:

Step-up FDR- 0.05 (Benjamini Hochberg); Fold change 2

Performed ANOVA and obtained DE mRNAs using create lists.

Performed GO enrichment- Enrichment Score- Higher the enrichment score, higher if the representation of that functional group in the gene list. Enrichment score is calculated by chi-square test comparing the proportion of a gene list in a group to the proportion of the background in our group. eg. A score of 1 means overexpressed functional category while a score of 3 means an even more significantly over-expressed category.

2. **Pathway analysis:**

Target genes and pathways for miRNAs DE at all three time points (56 in number) (made in IPA with DE miRNAs at all three months vs ctrl with < FDR 0.05)

Note: No DE miRNAs were observed in 7 months versus healthy control at step up FDR 0.05. Hence no data / miRNA list are included for this time point with adjusted p value. This also points out that at 7 months after infection has completely resolved in patients, there is no identifiable miRNA signature that can distinguish between normal and patient PBMCs.

Supplementary Figure 1. Hierarchical clustering –Heatmap showing relative intensity for 148 miRNAs in Patients at 1 month versus healthy controls, step up FDR 0.05

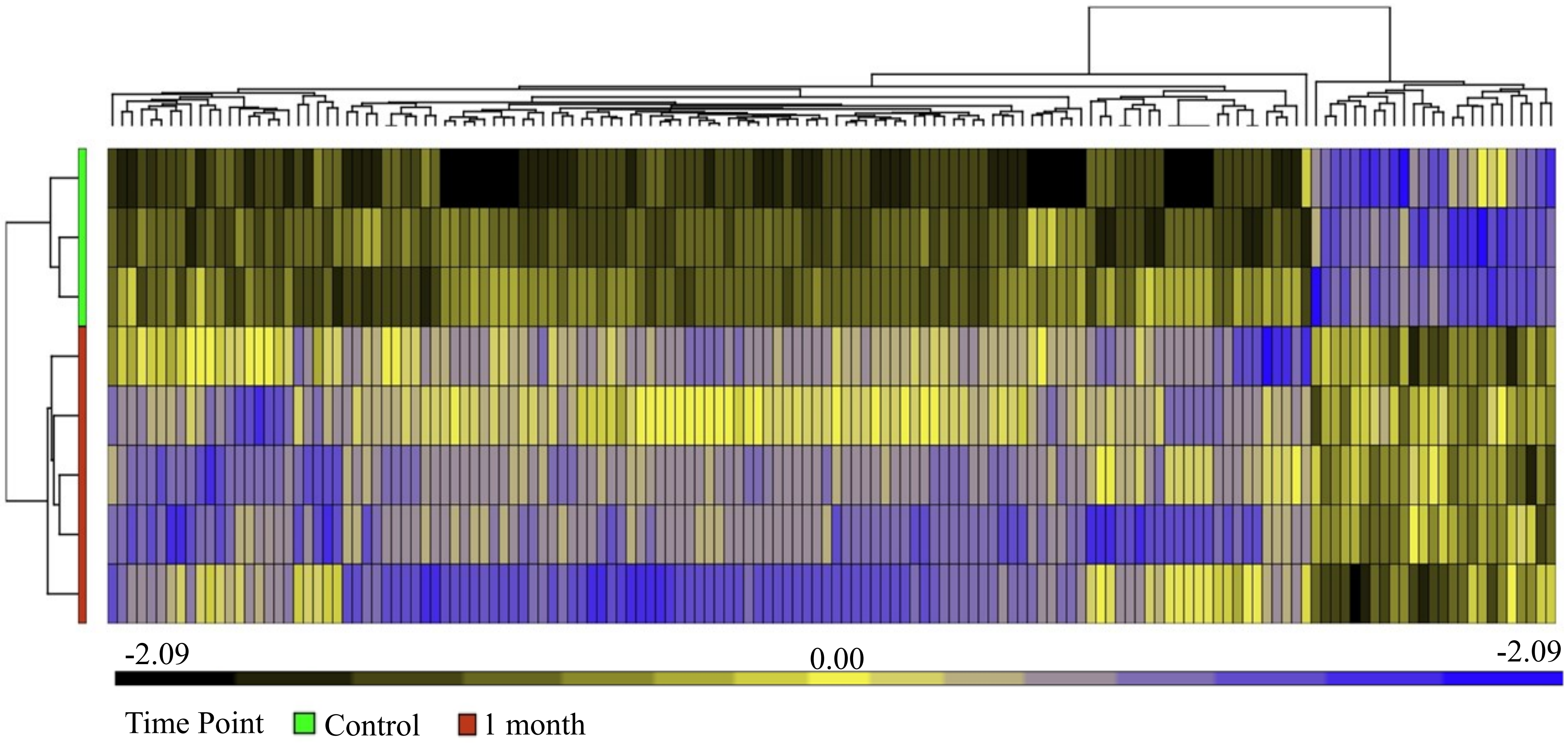

Supplementary Figure 2. Hierarchical clustering – Heatmap showing relative intensity for 68 miRNAs in Patients at 2 month versus healthy controls, step up FDR 0.05

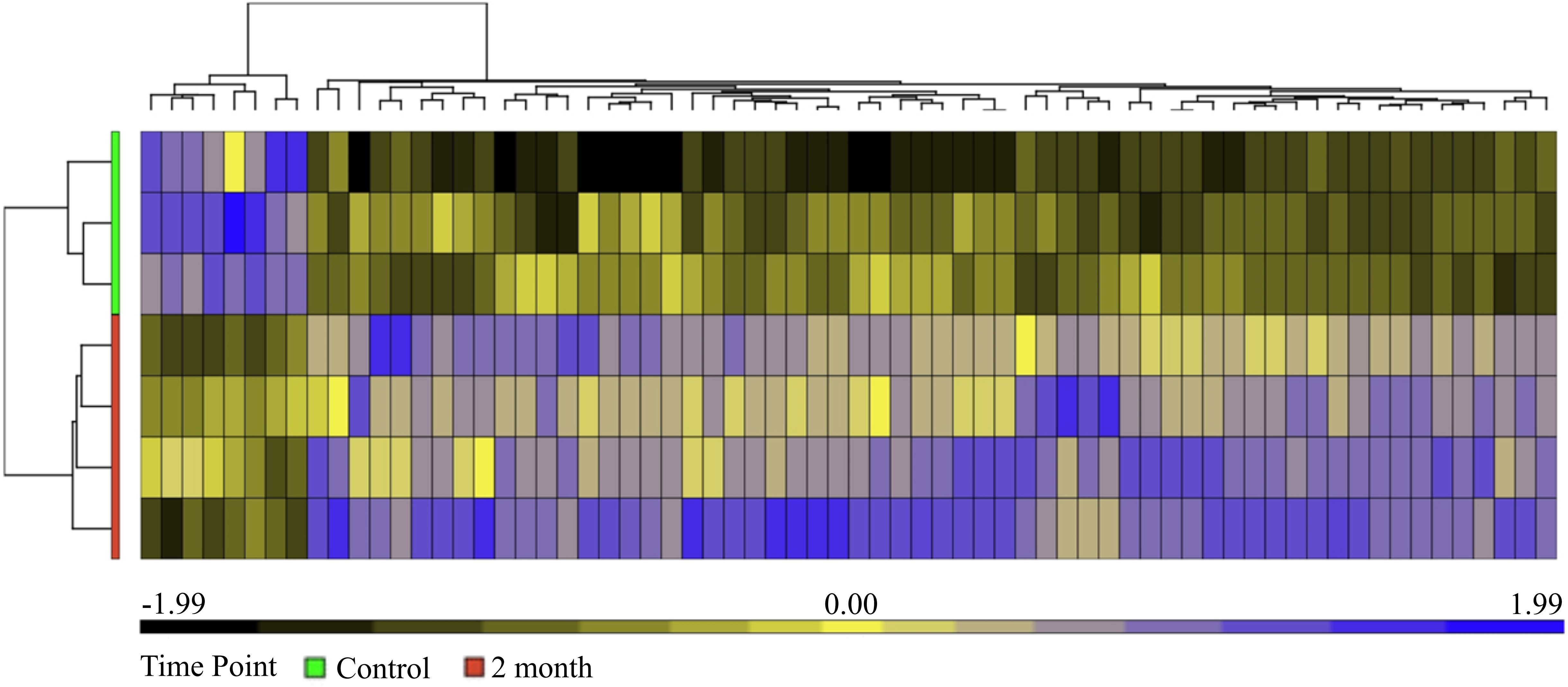

Supplementary Figure 3. Canonical Pathways for mRNA targets of DE miRNAs at Diagnosis

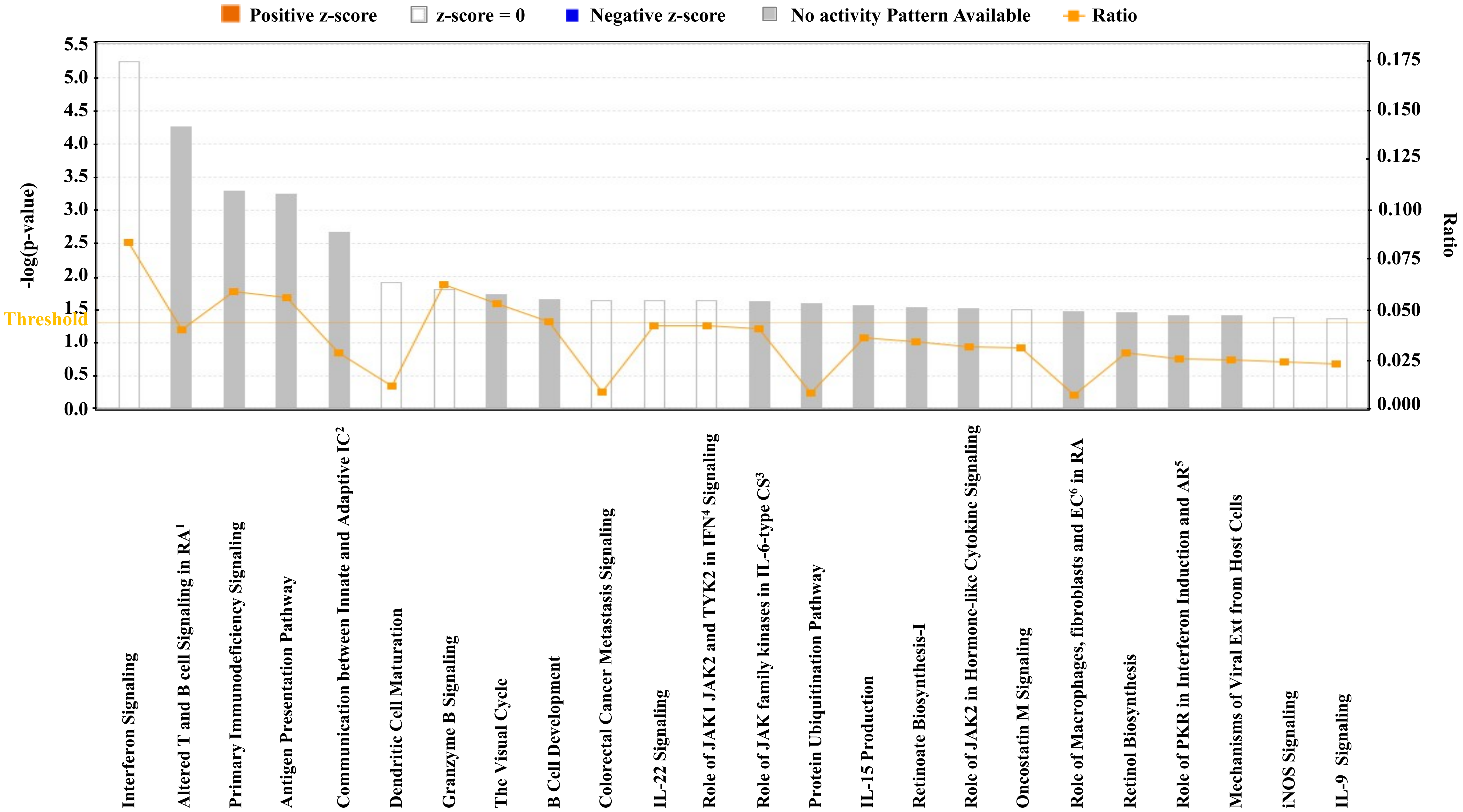

Supplementary Table S1. List of differentially expressed miRNAs present at three time points (Diagnosis, 1 month and 2 month); Adjusted FDR p value cut-off is 0.05

Time point ➞	Diagnosis		1 month		2 month	
DE miRNA ⬇	FoldChange	FDRp-value	FoldChange	FDRp-value	FoldChange	FDRp-value
ENSG00000239154	27.43	0.01	16.91	0.01	15.36	0.04
HBI-61	4.61	0.01	3.10	0.03	3.67	0.04
HBII-295	3.82	0.02	4.50	0.02	4.98	0.03
HBII-382	13.38	0.01	19.17	0.00	21.27	0.01
mgU2-25-61	13.38	0.01	19.17	0.00	21.27	0.01
hsa-mir-628	-2.08	0.02	-2.09	0.02	-2.12	0.05
hsa-mir-4281	8.29	0.01	10.77	0.00	6.76	0.02
hsa-mir-3687	16.46	0.01	14.37	0.01	15.64	0.01
hsa-mir-4539	3.55	0.01	2.39	0.03	3.02	0.02
hsa-mir-5095	12.49	0.01	12.29	0.01	8.26	0.04
hsa-miR-23b-3p	-2.30	0.02	-2.82	0.01	-2.29	0.05
hsa-miR-99b-5p	-4.34	0.01	-3.00	0.02	-3.93	0.01
hsa-miR-486-5p	-11.67	0.01	-13.07	0.01	-13.63	0.01
hsa-miR-628-3p	-4.83	0.01	-5.88	0.00	-5.64	0.01
hsa-miR-652-3p	-3.69	0.00	-2.50	0.00	-2.22	0.02
hsa-miR-23a-5p	-11.10	0.00	-11.92	0.00	-6.43	0.02
hsa-miR-149-3p	4.47	0.01	5.17	0.00	4.10	0.01
hsa-miR-744-5p	-3.96	0.00	-2.59	0.01	-2.41	0.04
hsa-miR-1207-5p	6.37	0.01	6.81	0.01	6.53	0.02
hsa-miR-1246	32.13	0.01	16.48	0.02	25.09	0.03
hsa-miR-1268a	6.83	0.01	7.86	0.01	5.44	0.05
hsa-miR-4270	8.80	0.01	5.92	0.02	6.48	0.04
hsa-miR-3687	45.57	0.00	48.50	0.00	41.32	0.01
hsa-miR-1268b	6.86	0.01	9.54	0.01	6.99	0.04
hsa-miR-4417	168.62	0.00	160.59	0.00	105.72	0.01
hsa-miR-4463	11.63	0.00	11.01	0.00	8.19	0.01
hsa-miR-4484	12.87	0.01	20.28	0.00	23.03	0.01
hsa-miR-4485	60.14	0.01	56.96	0.01	27.33	0.05
hsa-miR-4492	3.96	0.04	7.34	0.01	6.81	0.02
hsa-miR-4505	17.18	0.01	26.89	0.00	21.79	0.01
hsa-miR-4507	12.36	0.01	16.97	0.00	10.72	0.01
hsa-miR-4508	4.83	0.01	7.83	0.00	7.33	0.01
hsa-miR-4530	14.91	0.02	17.26	0.02	17.24	0.05
hsa-miR-4532	17.50	0.01	32.76	0.00	29.79	0.01
hsa-miR-1587	10.22	0.01	12.58	0.00	10.98	0.01
hsa-miR-4539	6.23	0.01	4.58	0.02	4.61	0.05
hsa-miR-3960	5.12	0.00	4.92	0.00	3.07	0.04
hsa-miR-4651	7.81	0.01	10.39	0.00	6.84	0.02
hsa-miR-4758-5p	11.44	0.01	11.54	0.00	7.09	0.04
hsa-miR-664b-5p	11.79	0.03	16.72	0.02	30.51	0.01
hsa-miR-937-5p	27.50	0.00	33.56	0.00	22.45	0.01
hsa-miR-3620-5p	5.30	0.01	8.36	0.00	5.25	0.04
hsa-miR-5787	3.58	0.02	5.35	0.01	4.28	0.03
hsa-miR-6085	11.81	0.01	13.08	0.01	9.78	0.03
hsa-miR-6126	11.05	0.01	16.37	0.00	16.75	0.01

hsa-miR-619-5p	25.50	0.01	31.27	0.00	21.19	0.01
hsa-miR-6743-5p	6.02	0.02	5.92	0.02	5.78	0.05
hsa-miR-6749-5p	14.10	0.00	16.38	0.00	10.59	0.01
hsa-miR-6752-5p	7.18	0.01	6.32	0.01	5.23	0.03
hsa-miR-6779-5p	11.86	0.01	15.37	0.00	10.95	0.01
hsa-miR-6798-5p	6.01	0.01	5.87	0.01	7.19	0.01
hsa-miR-6769b-5p	5.00	0.01	3.97	0.04	5.00	0.04
hsa-miR-6869-5p	11.56	0.01	10.03	0.01	6.64	0.04
hsa-miR-7107-5p	10.83	0.01	17.08	0.00	22.25	0.01
hsa-miR-7150	8.11	0.01	6.21	0.01	8.98	0.01
hsa-miR-4433b-3p	8.06	0.01	6.66	0.01	7.15	0.03

Supplementary Table S2. List of all differentially expressed miRNAs (unadjusted p value) at all four time points (Diagnosis, 1 month, 2 month and 7 month) Versus Healthy Control

Time Point ➞	Diagnosis		1 month		2 month		7 month	
miRNAs ⬇	Fold Change	Unadjusted p (ANOVA)	Fold Change	Unadjusted p (ANOVA)	Fold Change	Unadjusted p (ANOVA)	Fold Change	Unadjusted p (ANOVA)
ENSG00000207002	2.7103	0.0011	3.0421	0.0004	2.5414	0.0026		
ENSG00000238388	8.7357	0.0000	5.6177	0.0003	4.5200	0.0014	8.4907	0.0003
ENSG00000238388	5.9955	0.0002	4.6298	0.0006	3.6235	0.0034	6.9895	0.0005
ENSG00000238414	4.3939	0.0002	3.6758	0.0005	2.9697	0.0029	4.9414	0.0006
ENSG00000238549	2.0316	0.0009						
ENSG00000239080	2.5322	0.0008	2.3064	0.0018				
ENSG00000239154	27.4259	0.0000	16.9128	0.0001	15.3615	0.0002	34.1019	0.0001
ENSG00000239154	2.9339	0.0001	2.3359	0.0007			2.6533	0.0013
ENSG00000251940	2.6436	0.0001	2.4946	0.0001			2.3262	0.0018
ENSG00000263864	3.4962	0.0084	5.7438	0.0008	6.0948	0.0008	4.1238	0.0150
ENSG00000264086	3.4962	0.0084	5.7438	0.0008	6.0948	0.0008	4.1238	0.0150
ENSG00000264202	3.4962	0.0084	5.7438	0.0008	6.0948	0.0008	4.1238	0.0150
ENSG00000265651	3.0835	0.0046	3.4994	0.0021	4.7863	0.0005	2.8808	0.0238
ENSG00000265732	3.4962	0.0084	5.7438	0.0008	6.0948	0.0008	4.1238	0.0150
ENSG00000268890	2.6436	0.0001	2.4946	0.0001			2.3262	0.0018
HBI-43	2.0013	0.0071	2.2890	0.0021	2.8936	0.0004		
HBI-61	4.6105	0.0000	3.0966	0.0007	3.6657	0.0003		
HBII-115	4.6656	0.0032	4.7437	0.0029	9.6606	0.0002	5.1221	0.0092
HBII-142	3.3186	0.0417	4.0974	0.0196	12.1066	0.0005	5.3022	0.0258
HBII-295	3.8199	0.0006	4.4998	0.0002	4.9836	0.0002	2.3163	0.0426
HBII-296B			2.0408	0.0036	2.6099	0.0005		
HBII-382	13.3824	0.0000	19.1743	0.0000	21.2652	0.0000	7.8757	0.0014
mgU2-25-61	13.3824	0.0000	19.1743	0.0000	21.2652	0.0000	7.8757	0.0014
hsa-mir-320a	-2.2798	0.0014						
hsa-mir-628	-2.0777	0.0004	-2.0861	0.0004	-2.1197	0.0004		
hsa-mir-663a	3.7774	0.0011	2.8766	0.0060	2.5934	0.0144		
hsa-mir-885	3.0278	0.0001						
hsa-mir-1233-1	2.6751	0.0004						
hsa-mir-4281	8.2900	0.0000	10.7741	0.0000	6.7566	0.0001	5.1103	0.0022
hsa-mir-1233-2	2.6751	0.0004						
hsa-mir-3656	4.6640	0.0000	2.7046	0.0022	2.8156	0.0023	2.2471	0.0292
hsa-mir-3687	16.4618	0.0000	14.3660	0.0000	15.6428	0.0000	4.8622	0.0150
hsa-mir-4466	2.2819	0.0006	2.7172	0.0001	2.2067	0.0012		
hsa-mir-4539	3.5532	0.0000	2.3948	0.0005	3.0219	0.0001		
hsa-mir-4634	3.3002	0.0003						
hsa-mir-4758	2.3145	0.0006			2.1898	0.0015		
hsa-mir-4758	2.7012	0.0001			2.1810	0.0016	2.0071	0.0111
hsa-mir-5095	12.4920	0.0000	12.2937	0.0000	8.2629	0.0003		
hsa-mir-5703	3.7952	0.0001	3.6793	0.0001	2.9267	0.0011	2.8392	0.0051
hsa-mir-6089-1	4.6396	0.0001	4.1826	0.0001	3.3167	0.0009	3.3416	0.0032

hsa-mir-6800	5.0091	0.0002	3.8198	0.0011	2.5961	0.0145	2.5042	0.0414
hsa-mir-6800	5.1486	0.0001	4.1350	0.0005	2.6923	0.0094	2.5132	0.0345
hsa-mir-6894	2.0840	0.0004						
hsa-mir-7108	2.1639	0.0001	2.3665	0.0000				
hsa-mir-6089-2	4.6396	0.0001	4.1826	0.0001	3.3167	0.0009	3.3416	0.0032
hsa-mir-486-2	2.3462	0.0005						
hsa-let-7b-5p	-2.2306	0.0009						
hsa-let-7e-5p	-4.7895	0.0019	-7.0913	0.0003	-4.6994	0.0029	-3.4743	0.0297
hsa-miR-29a-3p	2.0208	0.0004						
hsa-miR-197-3p	-3.1906	0.0002	-2.8062	0.0007	-2.3701	0.0036	-2.3919	0.0106
hsa-miR-214-3p	3.1167	0.0010			2.1081	0.0205		
hsa-miR-15b-5p			-2.2914	0.0009				
hsa-miR-23b-3p	-2.2972	0.0004	-2.8207	0.0000	-2.2895	0.0006		
hsa-miR-130a-3p	-5.3329	0.0012	-8.7884	0.0001	-6.4917	0.0007	-8.2115	0.0011
hsa-miR-132-3p	-2.9349	0.0004	-2.1370	0.0060				
hsa-miR-145-5p	-5.0990	0.0098	-9.0144	0.0012	-6.2274	0.0063	-7.3535	0.0111
hsa-miR-185-5p	-2.4367	0.0000						
hsa-miR-320a	-2.6826	0.0007						
hsa-miR-99b-5p	-4.3385	0.0000	-2.9967	0.0003	-3.9260	0.0001	-3.2287	0.0010
hsa-miR-361-5p	-2.0972	0.0000						
hsa-miR-326	-2.6119	0.0016	-3.0853	0.0004			-3.4632	0.0012
hsa-miR-151a-3p	-4.9346	0.0011	-4.2882	0.0023	-5.1323	0.0013	-4.2167	0.0106
hsa-miR-345-5p	-3.3033	0.0002					-2.0405	0.0289
hsa-miR-423-3p	-2.4949	0.0000						
hsa-miR-484	-2.3214	0.0001	-2.6657	0.0000			-2.1491	0.0016
hsa-miR-486-5p	-11.6733	0.0001	-13.0691	0.0000	-13.6317	0.0000	-11.0956	0.0006
hsa-miR-491-5p	-3.3912	0.0005	-3.0180	0.0011	-2.4109	0.0078	-2.3185	0.0265
hsa-miR-146b-5p			2.0914	0.0005			2.0246	0.0038
hsa-miR-500a-3p	-2.6789	0.0008						
hsa-miR-584-5p	-7.4762	0.0194	-22.7073	0.0011	-14.3300	0.0049	-31.4244	0.0028
hsa-miR-628-3p	-4.8322	0.0001	-5.8777	0.0000	-5.6357	0.0000	-7.3853	0.0001
hsa-miR-638	3.7526	0.0005	3.8083	0.0004	2.2621	0.0187	2.9122	0.0113
hsa-miR-652-3p	-3.6891	0.0000	-2.5002	0.0000	-2.2198	0.0001	-2.0864	0.0010
hsa-miR-421	-3.8862	0.0092	-9.6305	0.0002	-3.1158	0.0300		
hsa-miR-23a-5p	-11.0972	0.0000	-11.9197	0.0000	-6.4316	0.0001	-10.6032	0.0001
hsa-miR-27a-5p	-9.8758	0.0010	-12.6225	0.0004	-4.2588	0.0250	-6.8982	0.0142
hsa-miR-31-3p			-2.1482	0.0007				
hsa-miR-149-3p	4.4672	0.0000	5.1661	0.0000	4.0994	0.0000	2.8585	0.0025
hsa-miR-193a-5p	-5.7558	0.0006	-3.8074	0.0049	-3.2493	0.0138	-4.0781	0.0140
hsa-miR-296-3p	2.2731	0.0014						
hsa-miR-151a-5p	-3.7686	0.0002	-2.9852	0.0011	-3.2753	0.0008	-2.5040	0.0160
hsa-miR-423-5p	-3.4277	0.0001	-2.0791	0.0046	-2.3268	0.0023		

hsa-miR-486-3p	-4.4825	0.0009	-6.9079	0.0001	-4.7210	0.0010	-4.9403	0.0030
hsa-miR-502-3p	-2.4755	0.0010						
hsa-miR-505-5p	-4.4697	0.0023	-7.5748	0.0002	-2.9921	0.0209	-3.1080	0.0409
hsa-miR-532-3p	-3.5290	0.0016	-3.5466	0.0015				
hsa-miR-455-3p	9.5188	0.0002	3.5689	0.0121	4.6211	0.0051	10.2409	0.0009
hsa-miR-92b-5p	6.9236	0.0004	4.1835	0.0045	4.3645	0.0050		
hsa-miR-574-5p	4.1246	0.0007	4.4179	0.0005	3.7753	0.0017		
hsa-miR-744-5p	-3.9602	0.0000	-2.5856	0.0001	-2.4101	0.0003	-2.3856	0.0014
hsa-miR-885-3p	4.3964	0.0001	2.1861	0.0108	2.0391	0.0228		
hsa-miR-665	6.0232	0.0015	2.9760	0.0317	4.0498	0.0111		
hsa-miR-940	2.0973	0.0006						
hsa-miR-1225-5p	4.1725	0.0011	3.5472	0.0028	3.9490	0.0021		
hsa-miR-320b	-2.7025	0.0005						
hsa-miR-1301-3p	-4.2078	0.0007	-5.6895	0.0001	-4.1334	0.0011	-4.1664	0.0039
hsa-miR-1202	4.7272	0.0008	2.4158	0.0294	3.0994	0.0101		
hsa-miR-1207-5p	6.3655	0.0001	6.8113	0.0001	6.5330	0.0001	5.3234	0.0014
hsa-miR-1246	32.1344	0.0000	16.4813	0.0003	25.0925	0.0001	7.2357	0.0183
hsa-miR-1268a	6.8274	0.0001	7.8563	0.0000	5.4437	0.0004	2.8565	0.0329
hsa-miR-1281	6.7953	0.0005	6.4779	0.0006	4.9421	0.0028	7.9779	0.0015
hsa-miR-1825	2.7850	0.0009					2.3119	0.0153
hsa-miR-1908-5p	2.4158	0.0010	2.6694	0.0004				
hsa-miR-1915-3p	9.1867	0.0001	6.6030	0.0003	3.7086	0.0063	4.9292	0.0056
hsa-miR-1972	14.2808	0.0003	12.5384	0.0004	12.0169	0.0007		
hsa-miR-1973	33.9498	0.0003	8.5242	0.0109	12.0358	0.0057		
hsa-miR-2110	-2.3332	0.0139	-3.3890	0.0012			-2.3283	0.0416
hsa-miR-762	3.7286	0.0005	4.7292	0.0001	3.0555	0.0024	2.4391	0.0271
hsa-miR-2861	5.4343	0.0002	5.3677	0.0002	3.0072	0.0085	3.5333	0.0109
hsa-miR-3141	6.4497	0.0003	5.6490	0.0005	5.8700	0.0006	2.9117	0.0441
hsa-miR-3162-5p	8.3846	0.0002	4.1592	0.0053	8.4140	0.0003	7.3084	0.0025
hsa-miR-3185	14.8632	0.0002	12.2213	0.0005	6.8736	0.0049	5.0616	0.0342
hsa-miR-3197	2.5786	0.0004	2.1780	0.0021	4.5478	0.0000	2.2476	0.0073
hsa-miR-4270	8.7993	0.0001	5.9200	0.0004	6.4830	0.0004	5.6756	0.0028
hsa-miR-4281	3.0849	0.0015	3.3864	0.0008	2.4170	0.0109		
hsa-miR-3619-5p	2.2525	0.0006						
hsa-miR-3648	5.2980	0.0007	4.5946	0.0014	4.6458	0.0019		
hsa-miR-3656	3.2453	0.0001	3.7799	0.0000	2.4991	0.0011	2.5943	0.0032
hsa-miR-3665	4.8748	0.0001	4.5777	0.0001	2.6668	0.0050	2.9461	0.0084
hsa-miR-3687	45.5664	0.0000	48.4959	0.0000	41.3205	0.0000	13.1839	0.0003
hsa-miR-1268b	6.8627	0.0002	9.5392	0.0000	6.9891	0.0003		
hsa-miR-4417	168.6230	0.0000	160.5870	0.0000	105.7150	0.0000	27.5190	0.0005
hsa-miR-4448	2.1496	0.0001						
hsa-miR-4459	6.2779	0.0016	4.3746	0.0072	5.8035	0.0030	4.1210	0.0303

hsa-miR-3135b	14.3150	0.0002	9.7659	0.0007	4.1622	0.0211		
hsa-miR-4463	11.6328	0.0000	11.0099	0.0000	8.1899	0.0000	5.7005	0.0011
hsa-miR-4466	3.7233	0.0005	4.2398	0.0002	3.0496	0.0028	2.2856	0.0419
hsa-miR-4484	12.8697	0.0001	20.2846	0.0000	23.0291	0.0000	13.7371	0.0005
hsa-miR-4485	60.1373	0.0001	56.9603	0.0001	27.3299	0.0006	7.3004	0.0431
hsa-miR-4492	3.9627	0.0015	7.3438	0.0001	6.8097	0.0001		
hsa-miR-4505	17.1811	0.0000	26.8906	0.0000	21.7879	0.0000	9.6651	0.0013
hsa-miR-4507	12.3649	0.0000	16.9748	0.0000	10.7166	0.0000	5.0084	0.0048
hsa-miR-4508	4.8306	0.0001	7.8336	0.0000	7.3333	0.0000	4.2058	0.0016
hsa-miR-4516	3.2859	0.0010	3.0304	0.0018	2.2162	0.0193		
hsa-miR-4530	14.9064	0.0005	17.2572	0.0003	17.2356	0.0005	7.1373	0.0200
hsa-miR-4532	17.4950	0.0001	32.7648	0.0000	29.7889	0.0000	20.0815	0.0004
hsa-miR-1587	10.2189	0.0000	12.5777	0.0000	10.9788	0.0000	3.2703	0.0248
hsa-miR-4539	6.2275	0.0001	4.5781	0.0004	4.6133	0.0005	2.4957	0.0429
hsa-miR-3940-5p	3.8357	0.0006	3.5689	0.0009	2.3130	0.0192	2.7386	0.0187
hsa-miR-3960	5.1171	0.0000	4.9242	0.0000	3.0692	0.0003	3.0906	0.0014
hsa-miR-4651	7.8117	0.0000	10.3908	0.0000	6.8439	0.0001	4.2361	0.0052
hsa-miR-4656	7.1931	0.0005	6.0829	0.0010	5.8227	0.0018		
hsa-miR-4665-5p	5.3346	0.0009	3.2036	0.0112	2.8972	0.0232		
hsa-miR-4687-3p	5.0312	0.0001	4.6621	0.0002	3.6065	0.0015	4.8220	0.0012
hsa-miR-4688	4.2328	0.0004	2.0816	0.0322				
hsa-miR-4689	8.3671	0.0001	4.7345	0.0010	4.5818	0.0017	3.4632	0.0196
hsa-miR-4690-5p	6.1155	0.0006	3.9089	0.0053	3.2679	0.0160		
hsa-miR-4695-5p	6.7660	0.0002	4.5447	0.0016	3.9492	0.0044	4.0152	0.0122
hsa-miR-4734	6.0430	0.0003	5.5875	0.0005	3.0156	0.0153	2.8369	0.0465
hsa-miR-4741	9.0965	0.0001	7.6773	0.0002	5.4258	0.0014	3.0537	0.0451
hsa-miR-4743-5p	2.8064	0.0018			2.5891	0.0043		
hsa-miR-4750-5p	3.4164	0.0010						
hsa-miR-4758-5p	11.4447	0.0000	11.5350	0.0000	7.0915	0.0003	4.7292	0.0064
hsa-miR-4763-3p	6.5589	0.0003	7.1061	0.0002	4.0198	0.0046	3.3637	0.0278
hsa-miR-4436b-5p	6.2078	0.0013	6.3728	0.0011	4.5452	0.0065		
hsa-miR-4787-5p	5.6991	0.0001	5.0949	0.0002	3.0336	0.0067	3.5507	0.0088
hsa-miR-5001-5p	12.6380	0.0000	8.8491	0.0001	5.0946	0.0019	5.9911	0.0035
hsa-miR-5189-5p	2.3701	0.0017						
hsa-miR-664b-5p	11.7940	0.0007	16.7237	0.0002	30.5079	0.0001	6.5194	0.0202
hsa-miR-5585-3p	3.3854	0.0008	2.3598	0.0099	2.0381	0.0330		
hsa-miR-937-5p	27.4976	0.0000	33.5643	0.0000	22.4506	0.0000	8.7404	0.0007
hsa-miR-1227-5p	3.6413	0.0010	3.3272	0.0018	2.5751	0.0118	2.4352	0.0389
hsa-miR-1229-5p	5.1765	0.0013	3.0228	0.0173	3.4551	0.0118		
hsa-miR-1233-5p	8.7938	0.0002	8.5114	0.0002	7.1563	0.0007		
hsa-miR-1237-5p	2.4911	0.0007	2.5383	0.0006				
hsa-miR-3620-5p	5.2996	0.0002	8.3578	0.0000	5.2525	0.0003	3.4601	0.0096

hsa-miR-4632-5p	9.5232	0.0001	11.2916	0.0001	6.8225	0.0008		
hsa-miR-5787	3.5815	0.0005	5.3533	0.0000	4.2838	0.0002	2.8679	0.0101
hsa-miR-6068	5.3336	0.0009	3.5044	0.0072				
hsa-miR-6085	11.8118	0.0000	13.0811	0.0000	9.7794	0.0001	3.5257	0.0310
hsa-miR-6086	2.9092	0.0015	2.0372	0.0205	3.8670	0.0003		
hsa-miR-6087	3.0695	0.0001	3.4169	0.0000	2.4183	0.0007	2.0769	0.0100
hsa-miR-6088	4.2400	0.0002	3.8997	0.0003	2.8683	0.0035	2.4865	0.0239
hsa-miR-6089	3.1523	0.0014	3.3528	0.0009	2.0518	0.0326		
hsa-miR-6090	3.6088	0.0006	3.4601	0.0008	2.1522	0.0243	2.3319	0.0351
hsa-miR-6125	6.1685	0.0002	5.4570	0.0004	3.2323	0.0080	3.5752	0.0140
hsa-miR-6126	11.0510	0.0001	16.3717	0.0000	16.7478	0.0000	5.2400	0.0110
hsa-miR-6132	6.4349	0.0009	9.7234	0.0002	7.5139	0.0007		
hsa-miR-6722-3p	8.2266	0.0001	8.6187	0.0001	6.0368	0.0008	3.4647	0.0280
hsa-miR-6723-5p	3.9204	0.0007						
hsa-miR-6724-5p	4.9614	0.0001	4.3941	0.0001	2.6068	0.0058	3.1556	0.0057
hsa-miR-504-3p	4.0975	0.0009	2.8793	0.0074	2.3933	0.0272		
hsa-miR-619-5p	25.4998	0.0000	31.2675	0.0000	21.1929	0.0000	5.3660	0.0178
hsa-miR-6727-5p	3.3683	0.0004	3.1505	0.0006			2.0523	0.0441
hsa-miR-6729-5p	5.6248	0.0002	5.3822	0.0002	3.1790	0.0056	3.6978	0.0080
hsa-miR-6741-5p	4.0662	0.0019	3.1032	0.0081	2.9868	0.0129		
hsa-miR-6743-5p	6.0204	0.0003	5.9211	0.0003	5.7792	0.0005		
hsa-miR-6746-5p	3.0751	0.0009	2.7573	0.0020	2.3004	0.0099		
hsa-miR-6749-5p	14.1028	0.0000	16.3810	0.0000	10.5948	0.0000	6.4893	0.0005
hsa-miR-6752-5p	7.1835	0.0000	6.3162	0.0000	5.2294	0.0001	3.7269	0.0041
hsa-miR-6754-3p	4.8643	0.0015	5.3917	0.0009	3.6055	0.0086		
hsa-miR-6756-5p	5.8857	0.0004	7.9492	0.0001	4.3084	0.0029		
hsa-miR-6763-5p	4.9733	0.0005	5.7417	0.0002	4.8192	0.0008		
hsa-miR-6774-5p	2.6452	0.0014						
hsa-miR-6775-5p	4.1186	0.0012	5.1692	0.0004	4.2338	0.0015	4.1354	0.0060
hsa-miR-6777-5p	2.0195	0.0019	2.0899	0.0013	2.0754	0.0020		
hsa-miR-6778-5p	11.0361	0.0012	5.7066	0.0111	10.8884	0.0018		
hsa-miR-6779-5p	11.8592	0.0000	15.3739	0.0000	10.9462	0.0000	6.1028	0.0023
hsa-miR-6787-5p	4.3934	0.0006	2.8447	0.0077	2.7204	0.0130		
hsa-miR-6791-5p	4.7996	0.0001	5.1719	0.0000	3.1461	0.0014	3.0816	0.0057
hsa-miR-6794-5p	7.3462	0.0000	5.6439	0.0001	4.3920	0.0006	3.9178	0.0043
hsa-miR-6798-5p	6.0089	0.0000	5.8662	0.0001	7.1927	0.0000	3.3172	0.0076
hsa-miR-6799-5p	6.2904	0.0002	4.2969	0.0017	3.4682	0.0069		
hsa-miR-6800-5p	11.8416	0.0002	10.5561	0.0002	5.1272	0.0060	6.4375	0.0081
hsa-miR-6802-5p	9.5756	0.0000	6.2937	0.0002	5.1353	0.0007		
hsa-miR-6803-5p	2.9769	0.0004	4.0340	0.0000	2.8072	0.0009	2.4455	0.0087
hsa-miR-6808-5p	3.2238	0.0005	2.9715	0.0009	3.1773	0.0008		
hsa-miR-6812-5p	6.5656	0.0007	3.8429	0.0079	5.3415	0.0024		

hsa-miR-6819-5p	7.2120	0.0002	3.8154	0.0051	4.6820	0.0026		
hsa-miR-6820-5p	7.4496	0.0004	5.6986	0.0014	4.9669	0.0035		
hsa-miR-6821-5p	7.0661	0.0002	7.4868	0.0002	4.1537	0.0038	4.8806	0.0062
hsa-miR-6858-5p	5.8505	0.0000	5.7113	0.0000	3.1664	0.0015	3.2954	0.0042
hsa-miR-6769b-5p	4.9963	0.0002	3.9652	0.0009	4.9990	0.0004		
hsa-miR-6860	3.0912	0.0014	2.2302	0.0139				
hsa-miR-6869-5p	11.5603	0.0000	10.0331	0.0000	6.6439	0.0004	8.3072	0.0007
hsa-miR-6879-5p	4.4032	0.0018	2.9442	0.0143	4.4747	0.0023		
hsa-miR-6880-5p	3.7398	0.0006			2.4785	0.0123		
hsa-miR-7107-5p	10.8291	0.0001	17.0816	0.0000	22.2493	0.0000	4.6720	0.0153
hsa-miR-7108-5p	4.5284	0.0002	4.5361	0.0002	2.7615	0.0071	3.3622	0.0072
hsa-miR-7111-5p	2.9685	0.0013			2.3229	0.0099		
hsa-miR-7150	8.1150	0.0000	6.2139	0.0000	8.9829	0.0000	2.4034	0.0429
hsa-miR-4433b-3p	8.0607	0.0001	6.6643	0.0001	7.1458	0.0002	6.1536	0.0014
hsa-miR-1273h-5p	12.8273	0.0008	9.3228	0.0023	13.7179	0.0010		
hsa-miR-8063	4.5580	0.0017						
hsa-miR-8069	4.8460	0.0014	4.5358	0.0019	2.5556	0.0404	3.0776	0.0400
hsa-miR-8072	2.8384	0.0006	2.8577	0.0006	2.0333	0.0123		
hsa-miR-8089	5.2545	0.0001	3.5865	0.0007	3.4347	0.0014		
SNORD121B	3.0269	0.0010	3.4226	0.0004	3.4024	0.0006		
SNORD121B	2.7848	0.0033	3.3209	0.0010	3.2238	0.0017		
U20					2.8984	0.0005		
U3	5.2330	0.0007	4.0019	0.0026	5.6308	0.0007	3.1684	0.0293
U3-2	3.7442	0.0010	3.3046	0.0021	4.2200	0.0007	2.4023	0.0442
U3-2B	3.7442	0.0010	3.3046	0.0021	4.2200	0.0007	2.4023	0.0442
U3-3	3.7442	0.0010	3.3046	0.0021	4.2200	0.0007	2.4023	0.0442
U3-4	3.7442	0.0010	3.3046	0.0021	4.2200	0.0007	2.4023	0.0442
U36C	3.0835	0.0046	3.4994	0.0021	4.7863	0.0005	2.8808	0.0238
U48	3.4962	0.0084	5.7438	0.0008	6.0948	0.0008	4.1238	0.0150
U65					2.0748	0.0005		
U88					2.3377	0.0000		
HBII-296B	2.1008	0.0027			2.5342	0.0007		
hsa-miR-642a-3p	2.1022	0.0021						
hsa-miR-4749-5p	4.9768	0.0021	3.6487	0.0088	4.4775	0.0046		
hsa-miR-5196-5p	2.5075	0.0111	2.5404	0.0102	3.7260	0.0013		
HBII-251	3.3632	0.0028	3.6489	0.0017	3.8047	0.0019		
E3	2.0414	0.0037			2.4632	0.0009		
hsa-miR-4728-5p	3.9529	0.0030	2.7214	0.0204	4.7290	0.0017		
hsa-miR-221-5p	2.2961	0.0026						
hsa-miR-6813-5p	6.9256	0.0020	6.0230	0.0034	7.2567	0.0023		
HBII-166	2.9057	0.0153	4.4581	0.0017	5.6603	0.0007	3.2646	0.0281
hsa-miR-1238-3p	2.6122	0.0024						

hsa-miR-6726-5p	4.9036	0.0061	5.8735	0.0030	4.6144	0.0102		
hsa-miR-6771-5p	5.3645	0.0023	5.0203	0.0031	3.7413	0.0146		
hsa-miR-328-5p	2.2212	0.0048	2.3249	0.0033				
hsa-miR-6870-5p	2.0805	0.0120			2.9491	0.0011		
hsa-miR-6848-5p	4.1192	0.0094	4.8156	0.0048	4.1413	0.0119		
hsa-miR-4284	2.3848	0.0412	2.8357	0.0174				
hsa-miR-5006-5p	2.7605	0.0020						
ENSG00000239055	2.1178	0.0025					2.7595	0.0014
ENSG00000221252	2.6706	0.0022	2.6190	0.0025	2.8666	0.0018		
hsa-miR-1251-3p	2.1676	0.0104	2.7037	0.0019	2.6936	0.0028	3.2311	0.0030
hsa-miR-1909-5p	4.0379	0.0029	3.1978	0.0095	4.4791	0.0024		
hsa-miR-532-5p	-2.4254	0.0026						
ACA41	2.4024	0.0063			2.6841	0.0039		
hsa-miR-4707-5p	3.7501	0.0022	2.7392	0.0128				
hsa-miR-6753-5p	3.5065	0.0028	2.1307	0.0469	2.2661	0.0408		
mgU6-53	2.0524	0.0076			2.5913	0.0015		
hsa-miR-4640-5p	3.5354	0.0023			2.8657	0.0103		
hsa-miR-6806-5p	2.0180	0.0068						
hsa-miR-6761-3p	2.2646	0.0049			2.2557	0.0067		
hsa-miR-4655-5p	2.4348	0.0087	2.1761	0.0185	2.3018	0.0163		
hsa-miR-197-5p	2.9271	0.0052			2.7995	0.0089		
hsa-miR-4745-5p	3.0796	0.0031	3.2165	0.0023				
hsa-miR-595	2.5552	0.0021						
U63	2.6821	0.0078	2.4028	0.0154	3.8630	0.0012		
U35A	6.0956	0.0101	6.2234	0.0095	12.8322	0.0013	9.8186	0.0095
hsa-miR-663a	2.1963	0.0044	2.2961	0.0030				
hsa-miR-6765-5p	2.3096	0.0055	2.3406	0.0050				
hsa-mir-4785	2.0334	0.0034						
hsa-miR-6846-5p	2.4276	0.0027						
hsa-miR-3196	2.9618	0.0030	2.5743	0.0074				
hsa-miR-143-3p	-3.3013	0.0264	-6.2861	0.0019	-5.6566	0.0040	-4.5829	0.0240
hsa-miR-1910-5p	5.0799	0.0063	5.6290	0.0042	4.7061	0.0111		
hsa-miR-425-3p	-2.7246	0.0026					-2.3612	0.0252
hsa-miR-6782-5p	4.2426	0.0066	3.9613	0.0089	5.5541	0.0028		
hsa-miR-6805-5p	4.2655	0.0020	3.8406	0.0035	3.0717	0.0144		
U91	2.8683	0.0380	2.9563	0.0336	6.8938	0.0013	4.4517	0.0211
mgU12-22-U4-8	2.8683	0.0380	2.9563	0.0336	6.8938	0.0013	4.4517	0.0211
hsa-mir-3648	3.9171	0.0034	3.8580	0.0037	2.9450	0.0187		
hsa-miR-6886-3p	3.8929	0.0031	2.4747	0.0321	2.5224	0.0357		
hsa-miR-4467	3.2326	0.0102	3.1110	0.0123				
U99	2.2725	0.0037	2.2939	0.0034	2.1715	0.0073		
hsa-miR-1228-5p	2.1467	0.0072	2.3217	0.0038				

hsa-miR-6816-5p	2.3362	0.0073	2.5655	0.0037				
hsa-miR-4667-5p	2.7640	0.0055			2.1743	0.0314		
hsa-miR-1273f	6.3680	0.0070	4.5464	0.0218	6.9825	0.0069		
hsa-miR-7975	4.2398	0.0057	4.3416	0.0051	3.9818	0.0099		
hsa-miR-4449	5.1450	0.0040	3.1730	0.0293	6.0019	0.0029		
hsa-miR-4726-5p	2.1132	0.0025						
HBII-419	5.7156	0.0062	4.4239	0.0159	9.0414	0.0017		
hsa-miR-4488	2.5463	0.0020	2.2690	0.0052				
ACA34	3.3000	0.0145			5.2608	0.0023		
hsa-miR-7114-5p	2.4492	0.0024			2.1042	0.0109		
hsa-miR-663b	10.4820	0.0025	5.2271	0.0217	9.7339	0.0043		
ENSG00000202252	4.3408	0.0151	3.7737	0.0253	9.0363	0.0014	4.9097	0.0309
hsa-miR-320c	-2.3642	0.0020						
hsa-miR-4498	2.2798	0.0058						
hsa-miR-382-5p	-7.0536	0.0046	-8.1284	0.0028	-4.5208	0.0262	-7.5242	0.0147
U94	2.8750	0.0150	2.3594	0.0408	4.6487	0.0017	3.0674	0.0337
hsa-miR-4685-5p	3.1115	0.0031						
hsa-miR-4669	2.1527	0.0049						
hsa-miR-6786-5p	2.4484	0.0058	2.2920	0.0093				
hsa-miR-7110-5p	7.3782	0.0053	6.1231	0.0098	6.4884	0.0106		
mgh18S-121	5.0242	0.0364			16.3418	0.0018	7.4993	0.0367
hsa-miR-182-5p	-3.7884	0.0072	-4.4778	0.0033	-4.2485	0.0057	-3.7060	0.0269
hsa-miR-4758-3p	3.3116	0.0050			3.4644	0.0053	2.9980	0.0287
hsa-mir-6850	2.2182	0.0041	2.0385	0.0084				
U46	6.5076	0.0082			9.6368	0.0031	6.0999	0.0323
HBII-336	2.4223	0.0067			2.6822	0.0044		
hsa-miR-1273d	4.1021	0.0100	5.1601	0.0038	4.3467	0.0103		
ENSG00000201009	4.0665	0.0090	3.9057	0.0107	4.9675	0.0052		
U46	4.0665	0.0090	3.9057	0.0107	4.9675	0.0052		
hsa-miR-139-5p	-7.3372	0.0117	-11.9676	0.0029	-6.8308	0.0183	-11.1794	0.0140
hsa-miR-6797-5p	2.7186	0.0158	2.6924	0.0166	3.2567	0.0078		
HBII-289	4.8529	0.0250	4.2178	0.0383	11.9319	0.0021	6.3113	0.0345
hsa-miR-574-3p	-4.2251	0.0063	-4.5253	0.0047	-2.7450	0.0497		
hsa-miR-454-3p	2.5973	0.0105						
hsa-miR-3180	5.7609	0.0040			3.1507	0.0494		
hsa-miR-4486	5.6412	0.0041			3.2405	0.0430		
hsa-miR-4298	5.6014	0.0041	4.0463	0.0149	5.8361	0.0048	4.3273	0.0355
hsa-miR-7704	2.0893	0.0169	2.6115	0.0033				
ENSG00000238686	2.1303	0.0028						
hsa-miR-93-3p	-2.7764	0.0173	-3.5001	0.0052				
U25	4.2310	0.0235	4.2822	0.0226	8.8882	0.0025	5.1915	0.0359
hsa-mir-139	-2.7690	0.0245	-3.6047	0.0068	-4.0366	0.0053	-4.0540	0.0151

hsa-miR-6510-5p	3.3378	0.0083			4.0522	0.0042		
hsa-miR-2392	3.4338	0.0060			3.0647	0.0139		
hsa-miR-200c-3p	-2.2165	0.0023						
hsa-miR-6850-5p	3.5683	0.0064	3.3281	0.0090				
hsa-miR-362-5p	-2.9463	0.0135						
hsa-miR-1231	4.4836	0.0048						
hsa-miR-675-5p	2.1899	0.0269						
hsa-miR-501-3p	-2.3541	0.0128	-2.3894	0.0116				
hsa-miR-6894-5p	2.1021	0.0036						
mgU6-53B	2.0121	0.0350	2.4061	0.0109	2.8410	0.0049		
hsa-miR-378a-3p	-2.1695	0.0039						
U58C	2.3296	0.0152			2.0419	0.0426		
hsa-mir-4674	2.0815	0.0098						
hsa-miR-3619-3p	3.1646	0.0063	2.1887	0.0466	2.8080	0.0156		
U46	6.3139	0.0125			9.7762	0.0044		
hsa-miR-6789-5p	3.3701	0.0055	2.7014	0.0179				
hsa-miR-1909-3p	4.3756	0.0070						
hsa-miR-7845-5p	3.0777	0.0056			2.8316	0.0119		
hsa-miR-4534	2.1711	0.0323			2.4537	0.0198		
U17a	2.2262	0.0300			3.2165	0.0046		
ACA7B	3.3927	0.0382			6.0541	0.0061	5.2114	0.0268
ACA7	3.3927	0.0382			6.0541	0.0061	5.2114	0.0268
ENSG00000206913	3.3927	0.0382			6.0541	0.0061	5.2114	0.0268
hsa-miR-1285-3p	4.6612	0.0063						
hsa-miR-6124	2.6141	0.0489			3.9224	0.0109		
hsa-miR-4732-5p	3.2541	0.0102						
hsa-miR-126-3p	-3.6690	0.0304	-5.7955	0.0057	-5.4583	0.0094		
hsa-miR-4634	4.2238	0.0123						
hsa-mir-4734	3.2517	0.0055			2.3272	0.0415		
U17a	2.0944	0.0265			2.7533	0.0058		
hsa-miR-3187-3p	2.8524	0.0091						
hsa-miR-6751-3p	3.4595	0.0101	2.5712	0.0402				
HBI-6	2.7500	0.0125	2.4285	0.0249	3.1836	0.0073		
hsa-miR-6737-5p	2.2300	0.0053						
U58C	2.2352	0.0229						
hsa-miR-4695-3p	4.0354	0.0083			3.0988	0.0319		
hsa-miR-5095	2.3275	0.0442	2.7505	0.0191				
hsa-miR-3937	2.7759	0.0118	2.1749	0.0448				
U60	2.0134	0.0378			2.8763	0.0051		
hsa-miR-3120-5p	2.5381	0.0148						
hsa-mir-4449	2.6459	0.0094	2.0504	0.0433				
hsa-miR-4674	3.0982	0.0122	2.7493	0.0221				

hsa-miR-554	3.4751	0.0052						
U29	3.4839	0.0260			5.1641	0.0074	3.8803	0.0483
hsa-miR-181a-2-3p	-3.6732	0.0244						
hsa-mir-6090	3.5826	0.0063						
U82	3.9413	0.0217			5.9081	0.0064		
hsa-miR-7641	3.5177	0.0464	6.4884	0.0058	4.8019	0.0207		
ACA32	2.8675	0.0186	3.3496	0.0086	3.2024	0.0139		
hsa-miR-1343-5p	3.4908	0.0113						
hsa-miR-4529-3p	2.0916	0.0232			2.1207	0.0264		
hsa-miR-5739	3.9596	0.0137			3.8792	0.0189		
hsa-miR-346	3.3239	0.0153	3.0600	0.0222				
hsa-miR-409-3p	-7.9747	0.0130	-9.4562	0.0082	-6.9360	0.0238		
hsa-miR-6790-5p	3.9426	0.0098						
hsa-miR-1290	2.1449	0.0158			2.1023	0.0229		
ACA32	2.9822	0.0160	3.0878	0.0135	3.3300	0.0121		
U49A	2.3073	0.0394			3.3905	0.0068		
hsa-mir-3960	2.0067	0.0142						
U34	3.4926	0.0481	4.0887	0.0287	6.9876	0.0062		
hsa-miR-3180-3p	4.9520	0.0102						
hsa-miR-142-5p	4.3918	0.0087			3.3972	0.0305		
hsa-miR-6716-5p	4.3128	0.0067			3.1013	0.0337		
hsa-miR-1247-3p	8.4725	0.0117			8.0805	0.0169		
hsa-miR-3609	7.2770	0.0450			15.2463	0.0119	13.6576	0.0360
hsa-miR-99a-5p	3.5156	0.0392	4.0539	0.0240	4.5798	0.0197	6.8943	0.0144
hsa-mir-6723	2.0464	0.0130						
hsa-miR-1538	2.0407	0.0428						
hsa-miR-7847-3p	5.6358	0.0184			6.2006	0.0176	5.9535	0.0450
hsa-miR-5010-5p	2.0115	0.0065						
hsa-miR-379-5p	-5.2121	0.0120	-4.8102	0.0158				
hsa-miR-4433-3p	3.7567	0.0089	2.6898	0.0394	3.2003	0.0230		
hsa-miR-6776-5p	2.4497	0.0121						
hsa-mir-6790	2.0675	0.0142						
hsa-miR-7109-5p	3.2745	0.0428	3.2259	0.0451	4.4856	0.0174		
mgU6-53	2.1703	0.0255			2.4944	0.0136		
mgh28S-2409	3.0660	0.0149	2.7404	0.0256	3.2645	0.0141		
hsa-miR-6889-5p	2.4541	0.0347			2.5930	0.0325		
hsa-miR-29b-3p	2.2143	0.0147	2.1296	0.0192				
hsa-miR-6824-5p	3.9467	0.0126						
ENSG00000207098	2.1903	0.0197						
U22	2.1426	0.0413	2.1576	0.0398	2.4785	0.0227		
U16	2.3951	0.0331	2.2609	0.0445	2.6945	0.0225		
hsa-mir-3910-1	-2.5225	0.0121			-2.3996	0.0207		

hsa-miR-6075	2.5512	0.0139				
ENSG00000252277	-3.2022	0.0231			-3.9153	0.0126
hsa-miR-4800-3p	3.0867	0.0127				
hsa-miR-8073	2.1050	0.0222				
HBII-85-29	2.6044	0.0391	2.5843	0.0405	3.7152	0.0099
hsa-miR-6893-3p	2.0365	0.0252				
ACA42	2.0694	0.0416			2.7780	0.0093
hsa-miR-3201	-2.4873	0.0138	-2.0659	0.0418	-2.4419	0.0196
ENSG00000263362	3.6440	0.0314			5.2985	0.0106
ENSG00000265156	3.6440	0.0314			5.2985	0.0106
ENSG00000265707	3.6440	0.0314			5.2985	0.0106
ENSG00000266300	3.6440	0.0314			5.2985	0.0106
U52	3.6440	0.0314			5.2985	0.0106
hsa-miR-432-5p	-6.6139	0.0234	-8.5329	0.0119		
hsa-miR-939-5p	2.9635	0.0124	2.3892	0.0374	2.7086	0.0248
hsa-miR-328-3p	-3.1782	0.0209	-3.5758	0.0124		
hsa-miR-6757-5p	3.1135	0.0216	2.6418	0.0441		
hsa-miR-24-2-5p	-2.8834	0.0351	-3.2932	0.0200		
hsa-miR-500a-5p	-2.7714	0.0493				
hsa-mir-320c-1	-2.0843	0.0177			-2.0414	0.0257
hsa-miR-4646-5p	4.0209	0.0434			5.4540	0.0215
ENSG00000238450	2.9678	0.0168	2.4821	0.0398	3.0186	0.0196
hsa-miR-3621	3.2281	0.0168	2.7060	0.0371		
ENSG00000238450	2.6638	0.0266	2.5519	0.0328	2.9039	0.0219
hsa-miR-3188	2.4402	0.0290				
hsa-miR-5100	2.0383	0.0368	2.0888	0.0316		
hsa-miR-5572	2.3012	0.0243				
hsa-miR-4750-3p	2.0039	0.0218				
hsa-miR-6795-5p	2.0567	0.0339	2.0184	0.0381		
hsa-miR-4649-5p	3.0187	0.0298				
hsa-miR-487b-3p	-5.1982	0.0443	-7.9502	0.0148		
hsa-miR-6845-5p	2.0737	0.0293	2.0805	0.0287		
hsa-miR-6781-5p	2.6008	0.0164			2.2130	0.0480
hsa-mir-663a	2.2339	0.0350	2.2858	0.0308	2.2610	0.0399
hsa-mir-4485	2.4013	0.0383				
hsa-miR-4454	2.4117	0.0242				
hsa-miR-8071	2.0660	0.0317				
hsa-miR-6882-3p	2.0791	0.0228				
hsa-miR-6861-5p	2.3756	0.0359	2.2750	0.0447		
HBII-95	2.7501	0.0417				
hsa-miR-6836-5p	2.3146	0.0439				
HBI-61	2.7121	0.0446	2.7613	0.0414		

hsa-mir-1248	2.7121	0.0446	2.7613	0.0414				
hsa-miR-3065-5p	2.5861	0.0243						
hsa-miR-129-5p	2.5075	0.0276						
snR38A	2.0612	0.0395						
hsa-mir-6858	2.2224	0.0385						
hsa-miR-451a			-9.7623	0.0029	-18.2213	0.0006	-17.0489	0.0030
ENSG00000207002			2.2838	0.0037				
hsa-miR-29b-1-5p			-4.1721	0.0025	-2.4171	0.0481	-5.1824	0.0045
HBII-142			4.2793	0.0239	12.8661	0.0008	5.7858	0.0282
hsa-miR-148b-3p			-2.4045	0.0329				
HBII-85-24			2.2013	0.0023				
hsa-miR-374b-5p			-4.6459	0.0066			-3.7925	0.0441
HBII-85-15			2.2006	0.0055	2.1894	0.0076		
U74			2.3741	0.0045	2.1196	0.0140		
hsa-miR-6891-5p			7.2428	0.0137	10.9848	0.0057		
hsa-miR-128-3p			-3.6521	0.0047				
ENSG00000266834			2.2497	0.0042				
U24			2.2497	0.0042				
hsa-mir-6746			2.1587	0.0087				
hsa-miR-199a-3p			-13.5678	0.0028	-6.3642	0.0276		
hsa-miR-199b-3p			-13.5678	0.0028	-6.3642	0.0276		
hsa-miR-125a-5p			-2.2053	0.0401	-3.1387	0.0074		
hsa-miR-30e-3p			-3.7803	0.0085				
HBII-316			2.2372	0.0139	2.7507	0.0045		
hsa-miR-199a-5p			-6.8067	0.0046			-5.9901	0.0248
hsa-miR-3679-5p			3.7134	0.0236	5.2317	0.0084		
hsa-mir-8075			2.1017	0.0252				
U101			3.4313	0.0162				
hsa-mir-8075			2.1068	0.0274				
SNORA38B			2.0442	0.0321	2.0209	0.0419		
hsa-let-7f-5p			-2.4259	0.0269				
U83B			3.3552	0.0366	5.7389	0.0066		
hsa-miR-3646			2.1887	0.0147				
U31			2.9160	0.0323	4.2802	0.0080		
hsa-miR-27b-3p			-4.2050	0.0057				
hsa-mir-711			4.5564	0.0072	3.1852	0.0377		
hsa-miR-335-5p			-2.1877	0.0082				
mgU6-53B			2.0856	0.0301	2.0811	0.0372		
HBII-429			2.1884	0.0318	2.8193	0.0092		
hsa-miR-4708-5p			2.5035	0.0211				
hsa-miR-3615			-2.8412	0.0080				
hsa-miR-4440			7.9008	0.0175				

hsa-miR-1184			2.8161	0.0384				
hsa-miR-6780b-5p			10.1525	0.0281	12.0032	0.0249		
hsa-mir-6869			3.3391	0.0214				
hsa-miR-4767			2.8457	0.0307				
hsa-miR-31-5p			2.2640	0.0144				
hsa-miR-4306			-2.4946	0.0447				
hsa-miR-223-3p			-3.2349	0.0137				
hsa-miR-483-5p			2.0522	0.0420	2.2143	0.0330		
hsa-mir-1299			5.3033	0.0382				
SNORA38B			2.9117	0.0346				
hsa-mir-4656			2.0629	0.0338				
hsa-miR-6877-3p			4.6120	0.0349				
hsa-mir-365a			2.0767	0.0355				
hsa-miR-30e-5p			2.0191	0.0307				
hsa-miR-92b-3p			-2.7738	0.0211				
hsa-miR-134-5p			-4.1599	0.0215				
hsa-miR-3124-5p			2.0386	0.0335				
hsa-miR-127-3p			-4.3116	0.0398				
hsa-miR-151b			-4.5824	0.0412				
U71d					2.1455	0.0017		
U90					2.3299	0.0007		
ACA6					2.5353	0.0017		
ACA5c					2.0545	0.0008		
ENSG00000252433					-2.1557	0.0018	-2.1207	0.0070
U106					2.0502	0.0039		
hsa-miR-6768-5p					2.1827	0.0136		
ACA61					3.4372	0.0014		
ACA16					2.8215	0.0043		
ACA16					2.6065	0.0050		
hsa-miR-660-5p					3.3808	0.0269		
U15A					2.4241	0.0065		
U105					3.7054	0.0032	3.2715	0.0177
ACA3					3.2439	0.0042		
gi:555853					2.1815	0.0033		
gi:555853					2.3162	0.0024		
hsa-miR-557					2.2178	0.0180		
HBII-135					8.8577	0.0027		
gi:555853					2.3795	0.0031		
hsa-miR-378d					2.7732	0.0234		
U38B					31.8246	0.0060		
U68					5.3630	0.0031		
U38B					25.1257	0.0052		

U53			6.2608	0.0029		
U97			2.8781	0.0034		
ENSG00000222345			2.5707	0.0046		
ACA48			4.0639	0.0056		
ACA8			3.3048	0.0248		
mgU6-77			2.9922	0.0052	2.5351	0.0341
ACA54			7.7157	0.0057		
hsa-miR-766-3p			-2.1442	0.0065		
HBII-239			3.0698	0.0098		
gi:555853			2.2399	0.0055		
U15B			2.8495	0.0044		
gi:555853			2.3769	0.0050		
hsa-miR-501-5p			2.2228	0.0333		
hsa-mir-3679			2.0689	0.0328		
hsa-miR-3607-5p			6.3386	0.0082		
U56			9.1595	0.0057		
gi:555853			2.2875	0.0059		
hsa-miR-3175			10.4096	0.0083		
ENSG00000207118			35.0283	0.0051		
gi:555853			2.2841	0.0063		
U26			6.6981	0.0052		
ACA34			2.6031	0.0085		
ENSG00000238936			17.7365	0.0064		
U85			3.4982	0.0106		
ACA27			3.6055	0.0063		
U27			3.6965	0.0116		
ENSG00000263442			6.0918	0.0114	5.0052	0.0478
ENSG00000264591			6.0918	0.0114	5.0052	0.0478
ENSG00000265325			6.0918	0.0114	5.0052	0.0478
ENSG00000265607			6.0918	0.0114	5.0052	0.0478
ENSG00000266646			6.0918	0.0114	5.0052	0.0478
ENSG00000266755			6.0918	0.0114	5.0052	0.0478
U84			6.0918	0.0114	5.0052	0.0478
gi:555853			2.1912	0.0070		
ENSG00000265941			3.5246	0.0061		
U36B			3.5246	0.0061		
U103B			2.3480	0.0429	3.2947	0.0209
U103			2.3480	0.0429	3.2947	0.0209
U27			3.7109	0.0119		
HBII-276			7.4059	0.0146	7.6270	0.0330
U47			4.6021	0.0096		
hsa-mir-3653			2.1178	0.0135		

SNORD125			2.1178	0.0135		
ACA10			2.5985	0.0123		
ENSG00000207187			2.5985	0.0123		
hsa-mir-326			2.0961	0.0154		
ACA40			3.4429	0.0403		
gi:555853			2.2168	0.0119		
U51			4.9811	0.0081		
U61			4.5374	0.0098		
U104			3.7627	0.0116	3.2261	0.0498
HBII-436			4.8742	0.0194		
U28			5.9136	0.0171		
U43			9.6144	0.0238		
U68			5.5116	0.0374		
gi:555853			2.1237	0.0100		
ACA18			3.5357	0.0135		
U28			7.2368	0.0205		
U43			9.6431	0.0255		
ACA20			4.5661	0.0316		
U56			8.4839	0.0107		
U95			5.1442	0.0147		
U59B			2.4720	0.0135		
U96a			2.8180	0.0221		
ENSG00000266284			4.1164	0.0150		
U36A			4.1164	0.0150		
U73a			4.9043	0.0184		
U49A			2.7515	0.0167		
ENSG00000212532			2.0533	0.0159		
U31			2.9917	0.0212		
hsa-miR-17-3p			3.5071	0.0364		
ACA49			2.3094	0.0327		
U54			5.5561	0.0245		
ENSG00000265706			3.8780	0.0219		
ENSG00000212532			2.1915	0.0285		
SNORD127			2.0490	0.0444		
ACA15			5.6107	0.0385		
ENSG00000206785			5.6107	0.0385		
ENSG00000207062			5.6107	0.0385		
U37			3.0216	0.0159		
U108			2.2383	0.0191		
U50			6.3190	0.0364		
U38A			8.8893	0.0210		
U75			8.6440	0.0461		

U21					2.4179	0.0244		
ACA48					4.1754	0.0223		
HBII-420					2.3677	0.0208		
snR39B					4.2874	0.0258		
HBII-210					3.5797	0.0212		
HBII-55					5.7875	0.0317		
ENSG00000252277					-4.8773	0.0275		
HBII-202					3.2952	0.0490	4.4338	0.0409
U83A					3.6256	0.0411		
U33					2.5243	0.0387		
mgh28S-2411					4.9014	0.0415		
hsa-miR-6127					3.8262	0.0291		
U17b					9.4889	0.0241		
hsa-miR-1273g-3p					2.6209	0.0424		
ACA44					2.4509	0.0489		
U105B					4.0021	0.0405		
HBII-85-26					-2.0878	0.0364		
ENSG00000201042					2.3216	0.0494		
SNORA38B					2.3216	0.0494		
hsa-mir-3154					3.4641	0.0423		
hsa-mir-4490							2.0346	0.0003
ENSG00000238775							2.3166	0.0058
hsa-miR-324-5p							-2.0039	0.0196
hsa-miR-3613-5p							8.9219	0.0239
hsa-miR-204-5p							2.0104	0.0209
hsa-miR-1273h-3p							-3.3214	0.0079
hsa-miR-1275							-2.4658	0.0390
hsa-miR-1228-3p							2.0806	0.0112
hsa-miR-629-5p							-4.7736	0.0107
hsa-miR-708-5p							4.3242	0.0408
U41							6.1554	0.0429
hsa-miR-1307-3p							-2.3337	0.0209
hsa-miR-331-3p							-3.6605	0.0377
hsa-miR-192-5p							2.0722	0.0175
hsa-miR-339-5p							-3.0729	0.0333
hsa-miR-671-3p							-2.6356	0.0375
hsa-miR-654-3p							-2.2904	0.0281
